# Metabolic Signatures of Adiposity in Young Adults: Mendelian Randomization Analysis and Effects of Weight Change

**DOI:** 10.1371/journal.pmed.1001765

**Published:** 2014-12-09

**Authors:** Peter Würtz, Qin Wang, Antti J. Kangas, Rebecca C. Richmond, Joni Skarp, Mika Tiainen, Tuulia Tynkkynen, Pasi Soininen, Aki S. Havulinna, Marika Kaakinen, Jorma S. Viikari, Markku J. Savolainen, Mika Kähönen, Terho Lehtimäki, Satu Männistö, Stefan Blankenberg, Tanja Zeller, Jaana Laitinen, Anneli Pouta, Pekka Mäntyselkä, Mauno Vanhala, Paul Elliott, Kirsi H. Pietiläinen, Samuli Ripatti, Veikko Salomaa, Olli T. Raitakari, Marjo-Riitta Järvelin, George Davey Smith, Mika Ala-Korpela

**Affiliations:** 1 Computational Medicine, Institute of Health Sciences, University of Oulu, Oulu, Finland; 2 Institute for Molecular Medicine Finland, University of Helsinki, Helsinki, Finland; 3 NMR Metabolomics Laboratory, School of Pharmacy, University of Eastern Finland, Kuopio, Finland; 4 MRC Integrative Epidemiology Unit, University of Bristol, Bristol, United Kingdom; 5 Department of Chronic Disease Prevention, National Institute for Health and Welfare, Helsinki, Finland; 6 Institute of Health Sciences and Biocenter Oulu, University of Oulu, Oulu, Finland; 7 Department of Medicine, University of Turku and Turku University Hospital, Turku, Finland; 8 Department of Internal Medicine, Clinical Research Center and Biocenter Oulu, University of Oulu and Oulu University Hospital, Oulu, Finland; 9 Department of Clinical Physiology, University of Tampere and Tampere University Hospital, Tampere, Finland; 10 Department of Clinical Chemistry, Fimlab Laboratories, University of Tampere, Tampere, Finland; 11 University Heart Center Hamburg, Hamburg, Germany; 12 Finnish Institute of Occupational Health, Helsinki, Finland; 13 Department of Obstetrics and Gynecology, Medical Research Center Oulu, Oulu University Hospital and University of Oulu, Oulu, Finland; 14 Department of Children, Young People and Families, National Institute for Health and Welfare, Oulu, Finland; 15 Primary Health Care, School of Medicine, University of Eastern Finland and Kuopio University Hospital, Kuopio, Finland; 16 Primary Health Care, Central Finland Central Hospital, Jyväskylä, Finland; 17 Department of Epidemiology and Biostatistics, MRC-PHE Centre for Environment and Health, Imperial College London, London, United Kingdom; 18 Department of Medicine, Helsinki University Central Hospital, Helsinki, Finland; 19 Research Programs Unit Diabetes and Obesity, University of Helsinki, Helsinki, Finland; 20 Wellcome Trust Sanger Institute, Hinxton, United Kingdom; 21 Hjelt Institute, Department of Public Health, University of Helsinki, Helsinki, Finland; 22 Research Centre of Applied and Preventive Cardiovascular Medicine, University of Turku, Turku, Finland; 23 Department of Clinical Physiology and Nuclear Medicine, Turku University Hospital, Turku, Finland; 24 Oulu University Hospital, Oulu, Finland; 25 Computational Medicine, School of Social and Community Medicine, University of Bristol, Bristol, United Kingdom; University Hospitals of Leicester NHS Trust, United Kingdom

## Abstract

**Background:**

Increased adiposity is linked with higher risk for cardiometabolic diseases. We aimed to determine to what extent elevated body mass index (BMI) within the normal weight range has causal effects on the detailed systemic metabolite profile in early adulthood.

**Methods and Findings:**

We used Mendelian randomization to estimate causal effects of BMI on 82 metabolic measures in 12,664 adolescents and young adults from four population-based cohorts in Finland (mean age 26 y, range 16–39 y; 51% women; mean ± standard deviation BMI 24±4 kg/m^2^). Circulating metabolites were quantified by high-throughput nuclear magnetic resonance metabolomics and biochemical assays. In cross-sectional analyses, elevated BMI was adversely associated with cardiometabolic risk markers throughout the systemic metabolite profile, including lipoprotein subclasses, fatty acid composition, amino acids, inflammatory markers, and various hormones (*p*<0.0005 for 68 measures). Metabolite associations with BMI were generally stronger for men than for women (median 136%, interquartile range 125%–183%). A gene score for predisposition to elevated BMI, composed of 32 established genetic correlates, was used as the instrument to assess causality. Causal effects of elevated BMI closely matched observational estimates (correspondence 87%±3%; *R* ^2^ = 0.89), suggesting causative influences of adiposity on the levels of numerous metabolites (*p*<0.0005 for 24 measures), including lipoprotein lipid subclasses and particle size, branched-chain and aromatic amino acids, and inflammation-related glycoprotein acetyls. Causal analyses of certain metabolites and potential sex differences warrant stronger statistical power. Metabolite changes associated with change in BMI during 6 y of follow-up were examined for 1,488 individuals. Change in BMI was accompanied by widespread metabolite changes, which had an association pattern similar to that of the cross-sectional observations, yet with greater metabolic effects (correspondence 160%±2%; *R* ^2^ = 0.92).

**Conclusions:**

Mendelian randomization indicates causal adverse effects of increased adiposity with multiple cardiometabolic risk markers across the metabolite profile in adolescents and young adults within the non-obese weight range. Consistent with the causal influences of adiposity, weight changes were paralleled by extensive metabolic changes, suggesting a broadly modifiable systemic metabolite profile in early adulthood.

*Please see later in the article for the Editors' Summary*

## Abstract 2

In this study, Wurtz and colleagues investigated to what extent elevated body mass index (BMI) within the normal weight range has causal influences on the detailed systemic metabolite profile in early adulthood using Mendelian randomization analysis.

*Please see later in the article for the Editors' Summary*

## Editors' Summary

**Background:**

Adiposity—having excessive body fat—is a growing global threat to public health. Body mass index (BMI, calculated by dividing a person's weight in kilograms by their height in meters squared) is a coarse indicator of excess body weight, but the measure is useful in large population studies. Compared to people with a lean body weight (a BMI of 18.5–24.9 kg/m^2^), individuals with higher BMI have an elevated risk of developing life-shortening cardiometabolic diseases—cardiovascular diseases that affect the heart and/or the blood vessels (for example, heart failure and stroke) and metabolic diseases that affect the cellular chemical reactions that sustain life (for example, diabetes). People become unhealthily fat by consuming food and drink that contains more energy (calories) than they need for their daily activities. So adiposity can be prevented and reversed by eating less and exercising more.

**Why Was This Study Done?:**

Epidemiological studies, which record the patterns of risk factors and disease in populations, suggest that the illness and death associated with excess body weight is partly attributable to abnormalities in how individuals with high adiposity metabolize carbohydrates and fats, leading to higher blood sugar and cholesterol levels. Further, adiposity is also associated with many other deviations in the metabolic profile than these commonly measured risk factors. However, epidemiological studies cannot prove that adiposity causes specific changes in a person's systemic (overall) metabolic profile because individuals with high BMI may share other characteristics (confounding factors) that are the actual causes of both adiposity and metabolic abnormalities. Moreover, having a change in some aspect of metabolism could also lead to adiposity, rather than vice versa (reverse causation). Importantly, if there is a causal effect of adiposity on cardiometabolic risk factor levels, it might be possible to prevent the progression towards cardiometabolic diseases by weight loss. Here, the researchers use “Mendelian randomization” to examine whether increased BMI within the normal and overweight range is causally influencing the metabolic risk factors from many biological pathways during early adulthood. Because gene variants are inherited randomly, they are not prone to confounding and are free from reverse causation. Several gene variants are known to lead to modestly increased BMI. Thus, an investigation of the associations between these gene variants and risk factors across the systemic metabolite profile in a population of healthy individuals can indicate whether higher BMI is causally related to known and novel metabolic risk factors and higher cardiometabolic disease risk.

**What Did the Researchers Do and Find?:**

The researchers measured the BMI of 12,664 adolescents and young adults (average BMI 24.7 kg/m^2^) living in Finland and the blood levels of 82 metabolites in these young individuals at a single time point. Statistical analysis of these data indicated that elevated BMI was adversely associated with numerous cardiometabolic risk factors. For example, elevated BMI was associated with raised levels of low-density lipoprotein, “bad” cholesterol that increases cardiovascular disease risk. Next, the researchers used a gene score for predisposition to increased BMI, composed of 32 gene variants correlated with increased BMI, as an “instrumental variable” to assess whether adiposity causes metabolite abnormalities. The effects on the systemic metabolite profile of a 1-kg/m^2^ increment in BMI due to genetic predisposition closely matched the effects of an observed 1-kg/m^2^ increment in adulthood BMI on the metabolic profile. That is, higher levels of adiposity had causal effects on the levels of numerous blood-based metabolic risk factors, including higher levels of low-density lipoprotein cholesterol and triglyceride-carrying lipoproteins, protein markers of chronic inflammation and adverse liver function, impaired insulin sensitivity, and elevated concentrations of several amino acids that have recently been linked with the risk for developing diabetes. Elevated BMI also causally led to lower levels of certain high-density lipoprotein lipids in the blood, a marker for the risk of future cardiovascular disease. Finally, an examination of the metabolic changes associated with changes in BMI in 1,488 young adults after a period of six years showed that those metabolic measures that were most strongly associated with BMI at a single time point likewise displayed the highest responsiveness to weight change over time.

**What Do These Findings Mean?:**

These findings suggest that increased adiposity has causal adverse effects on multiple cardiometabolic risk markers in non-obese young adults beyond the effects on cholesterol and blood sugar. Like all Mendelian randomization studies, the reliability of the causal association reported here depends on several assumptions made by the researchers. Nevertheless, these findings suggest that increased adiposity has causal adverse effects on multiple cardiometabolic risk markers in non-obese young adults. Importantly, the results of both the causal effect analyses and the longitudinal study suggest that there is no threshold below which a BMI increase does not adversely affect the metabolic profile, and that a systemic metabolic profile linked with high cardiometabolic disease risk that becomes established during early adulthood can be reversed. Overall, these findings therefore highlight the importance of weight reduction as a key target for metabolic risk factor control among young adults.

**Additional Information:**

Please access these websites via the online version of this summary at http://dx.doi.org/10.1371/journal.pmed.1001765.

- The Computational Medicine Research Team of the University of Oulu has a webpage that provides further information on metabolite profiling by high-throughput NMR metabolomics
- The World Health Organization provides information on obesity (in several languages)
- The Global Burden of Disease Study website provides the latest details about global obesity trends
- The UK National Health Service Choices website provides information about obesity, cardiovascular disease, and type 2 diabetes (including some personal stories)
- The American Heart Association provides information on all aspects of cardiovascular disease and diabetes and on keeping healthy; its website includes personal stories about heart attacks, stroke, and diabetes
- The US Centers for Disease Control and Prevention has information on all aspects of overweight and obesity and information about heart disease, stroke, and diabetes
- MedlinePlus provides links to other sources of information on heart disease, vascular disease, and obesity (in English and Spanish)
- Wikipedia has a page on Mendelian randomization (note: Wikipedia is a free online encyclopedia that anyone can edit; available in several languages)

## Introduction

The prevalence of overweight and obesity has reached epidemic proportions and represents a major threat to public health worldwide [1],[2]. Excess body weight, as assessed by body mass index (BMI), increases the risk for cardiovascular disease, type 2 diabetes, certain cancers, and premature death [2]–[6]. The increased morbidity and mortality linked with adiposity are partly attributed to abnormalities in glucose and lipid metabolism as well as hypertension [2],[3],[7]. Detailed metabolite profiling studies have further demonstrated global deviations in the molecular signatures of obesity when comparing small groups with large differences in body composition [8]–[10]. Yet it is unclear to what extent metabolic signatures of adiposity are observed in the systemic metabolism of adolescents and young adults within the non-obese range (BMI<30 kg/m^2^).

**Figure 1:**
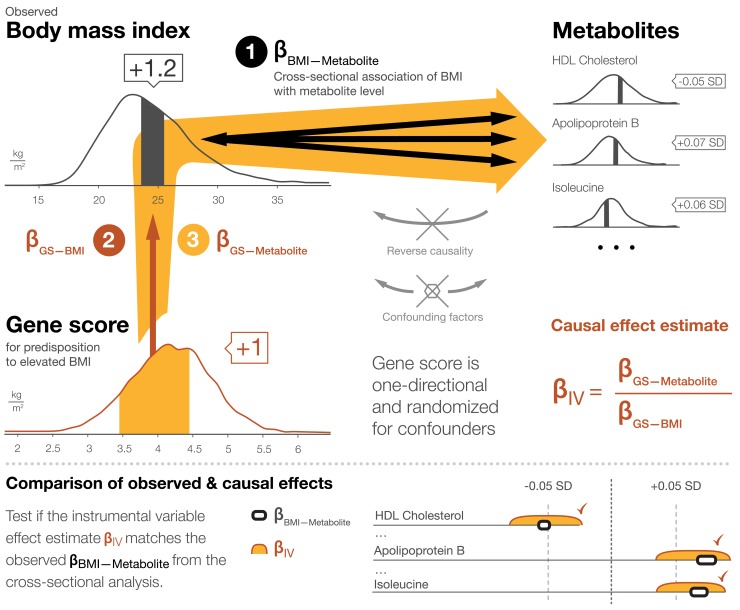
Mendelian randomization framework for estimating causal effects of BMI on the systemic metabolite profile. The principles of Mendelian randomization and core assumptions for the genetic instrument to be valid are detailed in Box 1.

The causal influence of adiposity on levels of metabolic risk markers can be examined within the framework of Mendelian randomization, an instrumental variable approach that uses genetic variation as an instrument to infer causality (Figure 1; Box 1) [11]–[13]. By analogy with the randomized controlled trial, the variation in adiposity caused by genotype assigns study participants to slightly different levels of BMI. The use of genetic variation as an instrument circumvents issues of confounding and reverse causation, which can otherwise distort observational study findings. The causal effects of elevated BMI on the metabolite profile can be quantified and compared to the correlations observed in traditional study designs [7],[14],[15]. Mendelian randomization studies have previously indicated causative influences of adiposity on dyslipidemia, hypertension, and insulin resistance by using common BMI-related genetic variants including the *FTO* (*fat mass and obesity associated*) gene as the instrumental variable [7],[14]–[17]. The cause-and-effect relationship between modestly elevated BMI and the detailed systemic metabolite profile in early adulthood, however, remains incompletely understood [7],[15].

**Box 1. Mendelian Randomization Framework for Estimating Causal Effects of BMI on the Systemic Metabolite Profile**

Mendelian randomization is an instrumental variable approach to infer causality in observational studies in the presence of potential confounding and reverse causation [11]–[13]. The modifiable exposure in this study is adiposity, as assessed by BMI. A gene score for predisposition to elevated BMI, composed of 32 allelic variants, is used as the instrument [34]. Causal effects of adiposity on metabolite levels are estimated by examining the gene score for association with observed BMI, as well as with each metabolic measure separately using a triangulation approach:

(1)The association between BMI and metabolite concentration is examined in a traditional cross-sectional study design (indicated schematically by the bi-directional black arrows in Figure 1; association magnitudes β_BMI-Metabolite_). The observational associations can arise from both directions: the metabolites could affect BMI, and BMI could affect the metabolite levels. These observational associations could also be generated, enhanced, or diminished because of confounding.

(2)The gene score is confirmed to be associated with BMI (dark red arrow in Figure 1; association magnitude β_GS-BMI_). All study participants carry some BMI-raising alleles, but those in the upper end of the distribution on average have a few units' higher BMI than those in the lower end. Association magnitudes for each cohort are listed in Table 1.

(3)The association between the gene score and each metabolite is tested (orange arrows in Figure 1; association magnitudes β_GS-Metabolite_). The genetic effect on the metabolites is assumed to be mediated entirely through the effect on BMI. Since genetic variants are assigned randomly at conception, transmission of the effects is independent of confounding factors. Further, genetic variation is not modified by phenotype (BMI or metabolite), so metabolite associations with the gene score are not affected by reverse causation. The gene score hereby serves to circumvent the limitations of the observational associations noted above. The instrumental variable (causal effect) estimate, β_IV_, is the gene score association with metabolite divided by the gene score association with BMI.

If adiposity exerts causal, non-confounded effects on a metabolite level, then the causal estimate is expected to be of a magnitude similar to that observed in the cross-sectional analysis. The causal estimate β_IV_ and the cross-sectional association β_BMI-Metabolite_ are compared using a *Z*-test for statistical difference.

The overall correspondence between the causal effects and observational associations are summarized in Figure 5 by the slope of the fit between the causal estimates and cross-sectional associations for the 82 metabolic measures analyzed. The cross-sectional associations and causal effect estimates in absolute concentration units and exact *p*-values are listed in Table S3.

The core assumptions for the instrument to be valid for estimating causal effects by Mendelian randomization are as follows:

- The gene score is robustly associated with observed BMI (Table 1).
- The gene score is independent of confounding factors. Associations of the gene score with age, sex, smoking, alcohol intake, physical activity, and socio-economic status are shown in Table S2.
- The gene score is related to the metabolite levels only through the effect on adiposity. Potential pleiotropy is assessed in Table 2, and association between the gene score and metabolites when adjusted for observed BMI are shown in Figure S3.
- All of the associations examined are linear and not affected by interactions. The linearity of the BMI–metabolite associations is illustrated in Figure S2. Whilst some sex differences were observed in cross-sectional analyses, the gene score had insufficient power to resolve such small potential differences in the causal estimates. Suggestive sex differences in the causal effect estimates are listed in Table S4.

**Table 1:** Characteristics of the study populations.

Clinical Characteristic	NFBC86	NFBC66	YFS	FINRISK 1997
Number of participants (women/men)	3,976 (1,997/1,979)	4,671 (2,321/2,350)	2,171 (1,155/1,016)	1,846 (995/851)
Age (y)	16 (—)	31 (—)	31.9 (4.9)	32.3 (4.5)
BMI (kg/m^2^)	21.2 (3.4)	24.6 (4.0)	25.0 (4.4)	24.7 (4.0)
Systolic blood pressure (mm Hg)	116 (13)	125 (13)	117 (13)	125 (14)
Total cholesterol (mmol/l)	4.2 (0.9)	5.3 (1.2)	5.0 (1.0)	5.0 (1.0)
HDL cholesterol (mmol/l)	1.5 (0.3)	1.7 (0.4)	1.6 (0.4)	1.6 (0.3)
Triglycerides (mmol/l)	0.9 [0.7–1.1]	1.0 [0.7–1.4]	1.1 [0.9–1.6]	0.9 [0.7–1.3]
Plasma glucose (mmol/l)	5.0 [4.7–5.2]	5.0 [4.7–5.3]	5.0 [4.7–5.3]	4.8 [4.5–5.1]
Insulin (IU/l)	9.5 [7.3–12]	7.5 [6.2–9.4]	6 [5]–[9]	4.7 [3.3–6.6]
Physical activity index (h/wk)	30 [18]–[43]	11 [4]–[20]	13 [3]–[31]	—
Alcohol usage (g/d)	—	4 [1]–[11]	5 [0–15]	4 [0–11]
Smoking prevalence (percent)	12% (11–13)	40% (39–42)	24% (22–26)	28% (26–30)
Prevalence of overweight (percent)	9% (8–10)	31% (30–32)	32% (30–33)	32% (30–34)
Prevalence of obesity (percent)	3% (2–3)	8% (7–9)	12% (11–13)	9% (8–10)
Association of gene score for elevated BMI with observed BMI (β ± standard error, kg/m^2^)	0.91±0.10	1.21±0.11	0.92±0.17	1.14±0.17
	*p* = 8×10^−21^	*p* = 1×10^−28^	*p* = 7×10^−8^	*p* = 6×10^−11^
	*F*-statistic = 88	*F*-statistic = 125	*F*-statistic = 29	*F*-statistic = 39
Variation in observed BMI explained by the gene score for elevated BMI	2.2%	2.6%	1.3%	2.1%

Values are mean (SD), median [interquartile range], or percentage (95% confidence interval) for normally distributed, skewed, and categorical variables, respectively. The gene score for predisposition to elevated BMI was derived based on weighting each genetic variant in the score by effects established previously in genome-wide meta-analysis [34].

**Table 2:** Genetic variants included in gene score for elevated BMI and pleiotropy assessment.

rs Number, Effect Allele/Other Allele	Nearest Gene	Weight in Gene Score	Effect Allele Frequency	Possible Proxy in NFBC86 (*) or in FINRISK (**)	Association with BMI in This Study, β (SE); *p*-Value	Correspondence between Causal and Observed Effect Estimates without Pertinent Variant in the Gene Score
rs1558902, A/T	*FTO*	0.39	39.9	rs1421085* (LD = 1.00)	0.110 (0.016); *p* = 1×10^−11^	Slope = 0.79±0.042
				rs9939609** (LD = 0.90)		Intercept = −0.0063
						*R* ^2^ = 0.82 [0.75–0.89]
rs2867125, C/T	*TMEM18*	0.31	84.3		0.063 (0.017); *p* = 0.0002	Slope = 0.87±0.034
						Intercept = −0.00076
						*R* ^2^ = 0.89 [0.85–0.93]
rs571312, A/C	*MC4R*	0.23	17.9		0.098 (0.016); *p* = 2×10^−9^	Slope = 0.87±0.036
						Intercept = −0.0017
						*R* ^2^ = 0.88 [0.83–0.93]
rs10938397, G/A	*GNPDA2*	0.18	50.4	rs1264198* (LD = 0.97)	0.043 (0.013); *p* = 0.0006	Slope = 0.88±0.039
						Intercept = −0.0028
						*R* ^2^ = 0.86 [0.81–0.91]
rs10767664, A/T	*BDNF*	0.19	82.7	rs2030323* (LD = 1.00)	0.041 (0.022); *p* = 0.06	Slope = 0.87±0.034
						Intercept = −0.003
						*R* ^2^ = 0.89 [0.85–0.93]
rs2815752, A/G	*NEGR1*	0.13	64.5		0.037 (0.019); *p* = 0.05	Slope = 0.82±0.033
						Intercept = 7.6×10^−5^
						*R* ^2^ = 0.88 [0.83–0.93]
rs7359397, T/C	*SH2B1*	0.15	41.6		0.033 (0.022); *p* = 0.10	Slope = 0.88±0.034
						Intercept = −0.0038
						*R* ^2^ = 0.89 [0.85–0.93]
rs9816226, T/A	*ETV5*	0.14	84.5	rs7647305* (LD = 0.72)	0.020 (0.017); *p* = 0.20	Slope = 0.85±0.036
						Intercept = −0.003
						*R* ^2^ = 0.87 [0.82–0.92]
rs3817334, T/C	*MTCH2*	0.06	39.7	rs10838738** (LD = 0.84)	0.020 (0.014); *p* = 0.20	Slope = 0.85±0.034
						Intercept = 0.00081
						*R* ^2^ = 0.89 [0.85–0.93]
rs29941, G/A	*KCTD15*	0.06	60.6	rs11084753** (LD = 0.65)	0.021 (0.013); *p* = 0.10	Slope = 0.9±0.033
						Intercept = −0.0023
						*R* ^2^ = 0.90 [0.86–0.94]
rs543874, G/A	*SEC16B*	0.22	17.7		0.100 (0.016); *p* = 3×10^−10^	Slope = 0.89±0.035
						Intercept = −0.0029
						*R* ^2^ = 0.89 [0.85–0.93]
rs987237, G/A	*TFAP2B*	0.13	20.4	Missing**	0.094 (0.017); *p* = 1×10^−8^	Slope = 0.84±0.037
						Intercept = −0.0021
						*R* ^2^ = 0.86 [0.81–0.91]
rs7138803, A/G	*FAIM2*	0.12	36.6		0.046 (0.013); *p* = 0.0005	Slope = 0.9±0.034
						Intercept = −0.0015
						*R* ^2^ = 0.90 [0.86–0.94]
rs10150332, C/T	*NRXN3*	0.13	23.1	rs17109256* (LD = 1.00)	0.052 (0.032); *p* = 0.10	Slope = 0.86±0.035
						Intercept = −0.0019
						*R* ^2^ = 0.88 [0.83–0.93]
rs713586, C/T	*RBJ*	0.14	42.9	rs10182181* (LD = 1.00)	0.056 (0.014); *p* = 4×10^−5^	Slope = 0.88±0.034
				Missing**		Intercept = −0.0025
						*R* ^2^ = 0.89 [0.85–0.93]
rs12444979, C/T	*GPRC5B*	0.17	87.5		0.061 (0.019); *p* = 0.001	Slope = 0.86±0.034
						Intercept = −0.0016
						*R* ^2^ = 0.88 [0.83–0.93]
rs2241423, G/A	*MAP2K5*	0.13	84.5		0.025 (0.017); *p* = 0.20	Slope = 0.87±0.034
						Intercept = −0.0027
						*R* ^2^ = 0.89 [0.82–0.92]
rs2287019, C/T	*QPCTL*	0.15	78.0	Missing**	0.020 (0.016); *p* = 0.20	Slope = 0.88±0.035
						Intercept = −0.004
						*R* ^2^ = 0.88 [0.83–0.93]
rs1514175, A/G	*TNNI3K*	0.07	48.9		0.052 (0.013); *p* = 3×10^−5^	Slope = 0.89±0.036
						Intercept = −0.00083
						*R* ^2^ = 0.88 [0.83–0.93]
rs13107325, T/C	*SLC39A8*	0.19	1.2		0.076 (0.058); *p* = 0.20	Slope = 0.86±0.035
						Intercept = −0.0019
						*R* ^2^ = 0.88 [0.83–0.93]
rs2112347, T/G	*FLJ35779*	0.10	60.3		0.023 (0.019); *p* = 0.20	Slope = 0.89±0.034
						Intercept = −0.0013
						*R* ^2^ = 0.89 [0.85–0.93]
rs10968576, G/A	*LRRN6C*	0.11	39.3		0.031 (0.016); *p* = 0.06	Slope = 0.86±0.036
						Intercept = −0.0022
						*R* ^2^ = 0.88 [0.83–0.93]
rs3810291, A/G	*TMEM160*	0.09	64.0		0.038 (0.014); *p* = 0.008	Slope = 0.86±0.034
						Intercept = −0.0017
						*R* ^2^ = 0.89 [0.85–0.93]
rs887912, T/C	*FANCL*	0.10	26.2		0.024 (0.014); *p* = 0.08	Slope = 0.87±0.035
						Intercept = −0.0019
						*R* ^2^ = 0.88 [0.83–0.93]
rs13078807, G/A	*CADM2*	0.10	16.0		0.059 (0.017); *p* = 0.0005	Slope = 0.87±0.034
						Intercept = −0.0021
						*R* ^2^ = 0.89 [0.85–0.93]
rs11847697, T/C	*PRKD1*	0.17	1.3	rs10134820* (LD = 0.74)	0.027 (0.054); *p* = 0.60	Slope = 0.86±0.034
						Intercept = −0.0022
						*R* ^2^ = 0.88 [0.83–0.93]
rs2890652, C/T	*LRP1B*	0.09	21.8	rs17834293* (LD = 0.70)	0.017 (0.025); *p* = 0.50	Slope = 0.87±0.035
						Intercept = −0.0018
						*R* ^2^ = 0.89 [0.85–0.93]
rs1555543, C/A	*PTBP2*	0.06	59.3	rs11165643* (LD = 1.00)	0.015 (0.013); *p* = 0.20	Slope = 0.87±0.034
						Intercept = −0.0023
						*R* ^2^ = 0.89 [0.85–0.93]
rs4771122, G/A	*MTIF3*	0.09	30.7	rs1006353* (LD = 0.74)	0.030 (0.015); *p* = 0.04	Slope = 0.86±0.034
				Missing**		Intercept = −0.0022
						*R* ^2^ = 0.88 [0.83–0.93]
rs4836133, A/C	*ZNF608*	0.07	48.7	rs6864049* (LD = 1.00)	−0.003 (0.020); *p* = 0.90	Slope = 0.86±0.034
						Intercept = −0.0022
						*R* ^2^ = 0.88 [0.83–0.93]
rs4929949, C/T	*RPL27A*	0.06	53.9	rs7127684* (LD = 1.00)	0.031 (0.016); *p* = 0.05	Slope = 0.87±0.034
						Intercept = −0.0022
						*R* ^2^ = 0.89 [0.85–0.93]
rs206936, G/A	*NUDT3*	0.06	22.7		0.026 (0.015); *p* = 0.07	Slope = 0.87±0.035
						Intercept = −0.0023
						*R* ^2^ = 0.89 [0.85–0.93]

Weights of the individual genetic variants are based on prior genome-wide analyses [34]. Allele frequencies are from the present study. Associations with BMI are linear regression coefficients in units of 1-SD increment in BMI per allele adjusted for age and sex, and meta-analyzed for the four cohorts. To assess the potential pleiotropic role of each genetic variant, we tested the effect of omitting each variant from the gene score by calculating the correspondence between cross-sectional associations and causal effect estimates.

LD, linkage disequilibrium; SE, standard error.

Intervention trials have shown favorable effects of weight reduction on cardiovascular risk factors [5],[18],[19]. Nevertheless, these trials have been conducted predominantly among clinically obese individuals [4],[5],[20]. The detailed metabolic effects of spontaneous weight change in healthy young adults remain incompletely investigated. Examining the responsiveness of the systemic metabolite profile to weight change could provide observational evidence on the anticipated effects of weight loss. To characterize the metabolic signatures of adiposity, we conducted comprehensive profiling of 12,664 adolescents and young adults from four population-based cohorts in Finland. The objectives were (1) to quantify cross-sectional associations of BMI with systemic metabolite levels, (2) to estimate causal effects of BMI on metabolite concentrations using Mendelian randomization, and (3) to assess the response of the metabolite profile to weight change during a 6-y follow-up. The pattern of metabolic aberrations observed cross-sectionally was compared to the causal effect estimates and longitudinal associations in order to summarize the influence of adiposity on the metabolic risk profile, and to clarify the metabolic consequences of weight change in early adulthood.

## Methods

### Study Populations

The study comprised four population-based cohorts (Table 1): the Northern Finland Birth Cohort of 1986 (NFBC86, *n* = 3,976 adolescents aged 16 y) [21], the Northern Finland Birth Cohort of 1966 (NFBC66, *n* = 4,671 individuals aged 31 y) [22], the Cardiovascular Risk in Young Finns Study (YFS, *n* = 2,171 individuals aged 24–39 y) [23], and FINRISK 1997 (*n* = 1,846 individuals aged 24–39 y) [24]. The study protocols were approved by the ethics committees of Northern Ostrobothnia Hospital District, Finland; the five universities with medical schools in Finland; and the National Public Health Institute, Helsinki, Finland. All participants gave written informed consent. Individuals aged 40 y or above were omitted from the present study (19% of eligible study population); this age cutoff was applied to focus on adolescent and young adults in order to minimize the influences of age and disease on metabolites and BMI [25]. Pregnant women (*n* = 208) and persons with diabetes or on anti-hypertensive or lipid treatment (*n* = 305) were also excluded from analyses. In total, 12,664 adolescents and young adults with measured comprehensive metabolite profiles and gene scores for predisposition to elevated BMI were included in the study. A subset of 1,488 persons from YFS further attended a 6-y follow-up survey, with the metabolite profile measured again. BMI was calculated as weight in kilograms divided by height in meters squared. Waist circumference, blood pressure, and standard biochemical assays were measured as part of the clinical examination. Smoking status, usage of alcohol, and physical activity index were assessed by questionnaires [26]. Secondary analyses were conducted for 2,850 older individuals from FINRISK as well as for the Pieksämäki Study (*n* = 628 individuals aged 40–57 y) [27]. Further details of the study populations are described in Text S1.

### Metabolite Quantification

A high-throughput serum nuclear magnetic resonance (NMR) spectroscopy platform [28] was utilized to quantify 67 metabolic measures that represent a broad molecular signature of the systemic metabolite profile. The metabolite set covers multiple metabolic pathways, and includes lipoprotein lipids, fatty acids, amino acids, and glycolysis precursors (Table S1). Fourteen lipoprotein subclasses were analyzed as part of the metabolite profile, with the subclass sizes defined as follows: extremely large very-low-density lipoproteins (VLDLs) (particle diameter from 75 nm upwards), five VLDL subclasses (average particle diameters of 64.0 nm, 53.6 nm, 44.5 nm, 36.8 nm, and 31.3 nm), intermediate-density lipoproteins (28.6 nm), three low-density lipoprotein (LDL) subclasses (25.5 nm, 23.0 nm, and 18.7 nm), and four high-density lipoprotein (HDL) subclasses (14.3 nm, 12.1 nm, 10.9 nm, and 8.7 nm). The NMR-based metabolite profiling employed in this study has previously been used in various epidemiological studies [25]–[31], and details of the experimentation have been described [28],[32],[33]. Furthermore, 15 additional measures, including various inflammatory markers, liver function surrogates, hormones, and blood pressure, were analyzed (Text S1). These additional metabolic measures, assayed in at least two of the cohorts, were selected to complement the comprehensive characterization of cardiometabolic effects of adiposity across multiple pathways and to enhance comparability with prior Mendelian randomization studies [7],[14]–[16].

### Genotyping

A gene score for predisposition to elevated BMI, composed of 32 single nucleotide polymorphisms firmly associated with BMI in prior genome-wide association studies, was used as the instrument to assess causality [34]. The genetic variants constituting the gene score are listed in Table 2. Genotyping of the 32 variants was conducted on Illumina HumanHap 370 k and 670 k platforms for NFBC66 and YFS, respectively. Variants not directly genotyped were imputed based on HapMap 2. Genotyping in NFBC86 was done using the Illumina Cardio-Metabochip [35]. For FINRISK, 28 out of the 32 variants were genotyped by iPLEX Sequenom MassARRAY. Genetic variants in high linkage disequilibrium were used in case of missing variants in the gene score, as listed in Table 2. For the instrumental variable analyses, the genotypes were combined into a gene score for elevated BMI by summing the allele count for each individual variant weighted by the effect size determined in large-scale genome-wide association meta-analyses [34].

### Statistical Analyses

Metabolites with skewed distributions were log-transformed prior to analyses. All metabolite concentrations were scaled to standard deviation (SD) units separately in each cohort. This scaling enabled comparison of association magnitudes across the metabolic measures. We calculated that 33 principal components explain>95% of the variance in each cohort [30], because of the correlation of the metabolic measures (Figure S1). With cross-sectional instrumental variable and longitudinal analyses performed, we used multiple testing correction for 100 independent tests according to the Bonferroni method. Statistical significance was therefore inferred at *p*<0.0005.

For cross-sectional analyses, linear regression models were fitted for each metabolite, with BMI as the explanatory variable and the metabolite concentration as outcome. The regression coefficients β_BMI–Metabolite_ were calculated in units of 1-SD metabolite concentration per 1-kg/m^2^ increment in BMI. Associations were adjusted for sex and age, if applicable. Results were analyzed separately for the four cohorts and combined using random effect inverse-variance-weighted meta-analysis [7]. The continuous shapes of the cross-sectional associations were illustrated by fitting quadratic curves of median and interquartile metabolite concentrations for each percentile of BMI for women and men (Figure S2). Significant sex interactions were observed for numerous metabolites in cross-sectional analyses; however, the absolute differences were mostly small. The sex differences were generally not resolved in the Mendelian randomization analyses, and the causal effects were therefore estimated for women and men combined.

For Mendelian randomization analyses, we used a gene score for predisposition to higher BMI as the instrumental variable. The individual genetic variants and their weights in the gene score are listed in Table S2. The Mendelian randomization framework for estimating causal effects and the core conditions for the gene score to serve as a valid instrument are described in Figure 1 and Box 1 [11]–[13]. Causal effect estimates and corresponding standard errors of BMI on the metabolic measures were calculated using the two-stage least squares method with adjustment for sex and age. The instrumental variable (causal effect) estimates are hereby equal to β_IV_ = β_GS-Metabolite_/β_GS-BMI_, where β_GS-Metabolite_ is the association of the gene score with the metabolic measure, and β_GS-BMI_ is the association of the gene score with observed BMI [12]. The causal estimates were computed separately for each cohort and subsequently meta-analyzed. The magnitudes of the causal effects were then compared to the corresponding cross-sectional observations: for each metabolite, we tested for statistical difference between the cross-sectional and instrumental association magnitudes using a classical *Z*-statistic [7],[14]. The overall correspondence between the association patterns was quantified by a linear fit of causal effect estimates versus cross-sectional associations. For the metabolites with suggestive sex interaction in association with the gene score, the Mendelian randomization analyses were further tested separately for women and men.

Changes in metabolite levels with changes in BMI over time were examined for 1,488 individuals for whom metabolite data were also available at 6-y follow-up. Longitudinal associations were assessed for each metabolite using a linear regression model with the 6-y change in BMI as predictor and the 6-y change in metabolite concentration as the outcome. The models were adjusted for age and sex. No robust sex interactions were observed in longitudinal analyses. To enable comparison with the cross-sectional associations, longitudinal association magnitudes are reported as the change in metabolite concentration (in units of baseline SD) per unit change in BMI during follow-up. Longitudinal association magnitudes were then tested for statistical difference from the corresponding cross-sectional associations in the full study population. The overall correspondence between the association patterns was quantified by a linear fit of longitudinal versus cross-sectional associations. The longitudinal analyses were additionally replicated for 1,372 individuals at 10-y follow-up in YFS, as well as in the Pieksämäki Study, consisting of 456 middle-aged persons with 6-y follow-up (Text S1) [27]. To further illustrate the metabolic changes paralleled by weight change, the median changes in metabolite concentrations were calculated for subgroups of people who displayed 3%–6% and 6%–10% weight loss or weight gain during the 6-y follow-up period in YFS. For these analyses, weight and metabolite concentrations were mean-centered for both baseline and follow-up to account for potential batch effects. The confidence intervals of the medians were estimated by 10,000 bootstrap replicates.

## Results

The study comprised 12,664 adolescents and young adults from four general population cohorts who all had detailed metabolite profiles measured and information on gene score for predisposition to elevated BMI. In addition, 1,488 individuals also had metabolite profiling data at 6-y follow-up. Clinical characteristics of the four cohorts are shown in Table 1. The mean BMI of 24.7 kg/m^2^ in the three cohorts of young adults reflects the population average in Finland [24]. Twenty-three percent were overweight, and 7% were obese. Mean concentrations of the assayed metabolites are listed in Table S1. The correlations between the metabolites are shown in Figure S1.

### BMI and the Systemic Metabolite Profile

**Figure 2:**
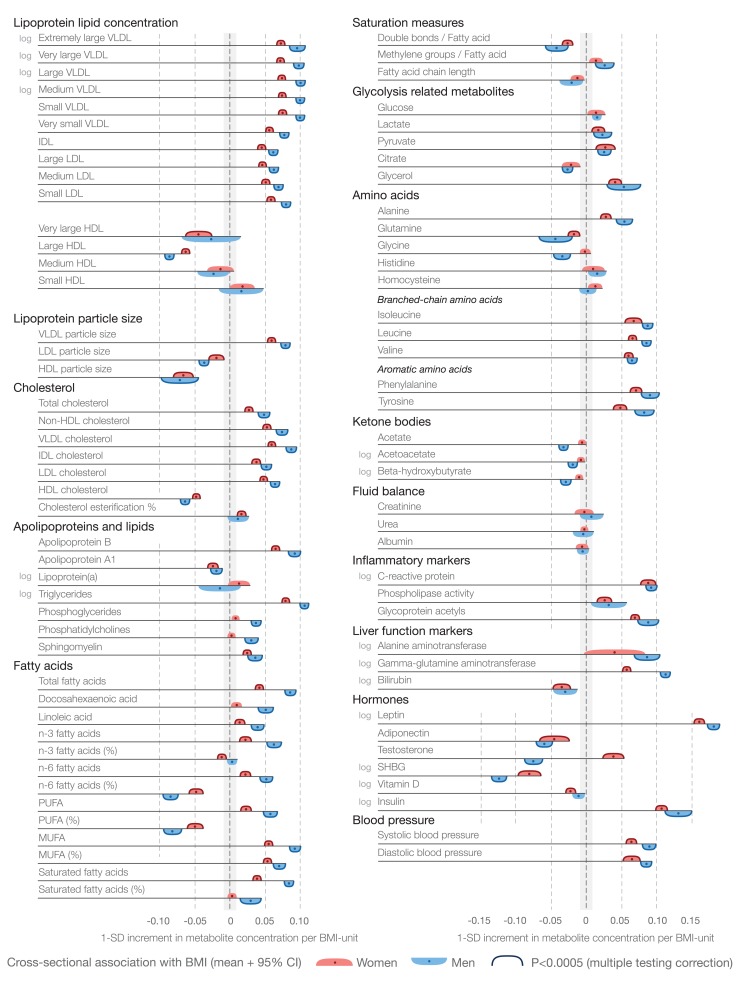
Cross-sectional associations of BMI with systemic metabolites for 6,468 women (red) and 6,196 men (blue). Association magnitudes are indicated in units of 1-SD metabolite concentration per 1-kg/m^2^ increment in BMI. Associations were adjusted for age and meta-analyzed for the four cohorts of adolescents and young adults. Colored dots indicate β-regression coefficients, colored shading denotes 95% confidence intervals, and boundaries on the shading indicate *p*<0.0005. The continuous shape of the associations and magnitudes in absolute concentration units are illustrated in Figure S2. MUFA, monounsaturated fatty acid; PUFA, polyunsaturated fatty acid; SHBG, sex hormone–binding globulin.

Cross-sectional associations of BMI with 82 metabolic measures are illustrated in Figure 2 for women (red) and men (blue). For both sexes, the majority of the metabolites were associated with BMI (66 measures for men and 61 for women at *p*<0.0005 in meta-analyses). Metabolite associations tended to be stronger for men than for women (median 136%, interquartile range 125%–183%); however, the association patterns were generally in the same direction, towards a more risk-prone systemic metabolite profile for those with higher BMI. Lipoprotein lipids displayed a characteristic association pattern with BMI: the most pronounced associations were observed for VLDL lipids, whereas associations with LDL lipids were weaker, albeit increased for small LDL lipid concentration. HDL lipids displayed heterogeneous associations, with strong inverse associations for large HDL lipid concentration and HDL particle size. Prominent direct associations with BMI were observed for monounsaturated and saturated fatty acids. The ratio of polyunsaturated fatty acids to total fatty acids was inversely associated with BMI. Fatty acid associations were about twice as strong for men as for women. Only weak associations were observed for glycolysis-related metabolites for both sexes in this young study population. In contrast, branched-chain and aromatic amino acids were strongly positively associated with BMI, with magnitudes comparable to those of total cholesterol and triglycerides. Further, sizeable associations were observed between BMI and metabolic risk factors such as markers of inflammation and liver function, as well as between BMI and adiposity-related hormones.

**Figure 3:**
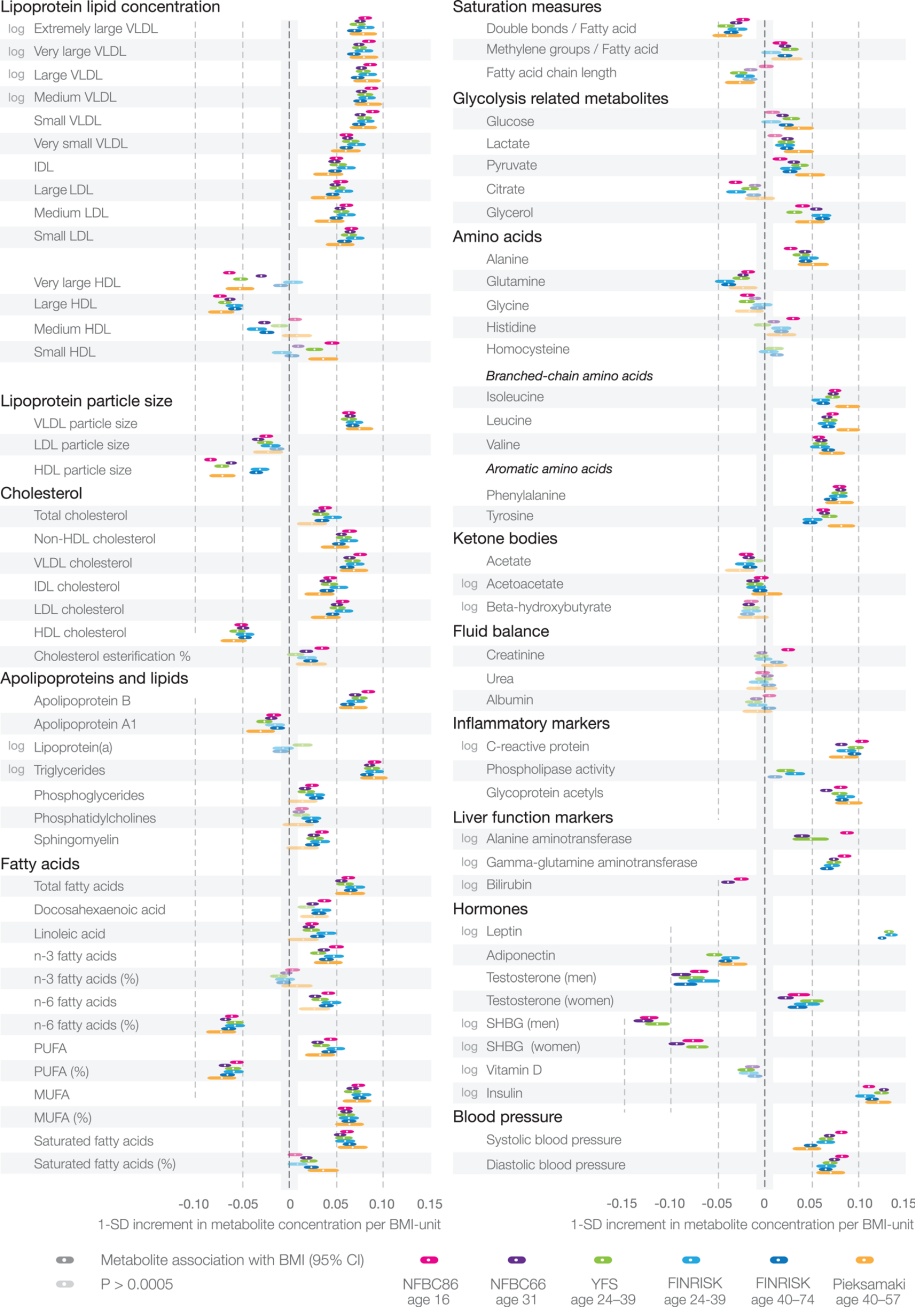
Cross-sectional associations of BMI with systemic metabolites across four cohorts of adolescents and young adults, and consistency in two populations of older individuals. Association magnitudes are in units of 1-SD metabolite concentration per 1-kg/m^2^ increment in BMI. Color coding indicates the respective cohorts. White dots indicate β-regression coefficients, colored shading indicates 95% confidence intervals, and darker shading denotes *p*<0.0005. The associations were analyzed for men and women combined and adjusted for sex and age, as well as smoking status, alcohol intake, and physical activity index. MUFA, monounsaturated fatty acid; PUFA, polyunsaturated fatty acid; SHBG, sex hormone–binding globulin.

Most metabolite associations followed approximately linear shapes across the range of BMI, with increases observed already within the normal weight range (BMI<25 kg/m^2^), as illustrated in Figure S2. Cross-sectional association magnitudes in absolute concentration units (e.g., mmol/l per kg/m^2^) are also indicated in Figure S2. The cross-sectional metabolite associations with BMI were coherent across all four study populations, as well as for middle-aged persons from the FINRISK study (*n* = 3,676, aged 40–74 y) and the Pieksämäki Study (*n* = 628, aged 40–57 y), and were similar when adjusting for smoking status, alcohol intake, and physical activity index (Figure 3).

### Causal Effects of Adiposity on the Metabolic Profile

The causal effects of BMI on the systemic metabolite profile were analyzed using Mendelian randomization. The principles of this instrumental variable framework are detailed in Box 1 [11]–[13]. A weighted gene score, composed of 32 established genetic variants for predisposition to elevated BMI (Table 2), served as the instrument to estimate causal metabolic effects per 1-kg/m^2^ increment in BMI [34]. The gene score was robustly correlated with observed BMI (Pearson's correlation *r* = 0.15; *p* = 2×10^−62^, *F*-statistic = 194) and explained 1.3%–2.6% of the variation in BMI in the study populations (Table 1). Further, the gene score was not associated with potential confounders including sex, age, smoking, alcohol usage, or physical activity, as assessed by questionnaires (Table S2). The Mendelian randomization analyses were combined for women and men, since there was limited evidence for sex interactions resolved by the genetic instrument.

**Figure 4:**
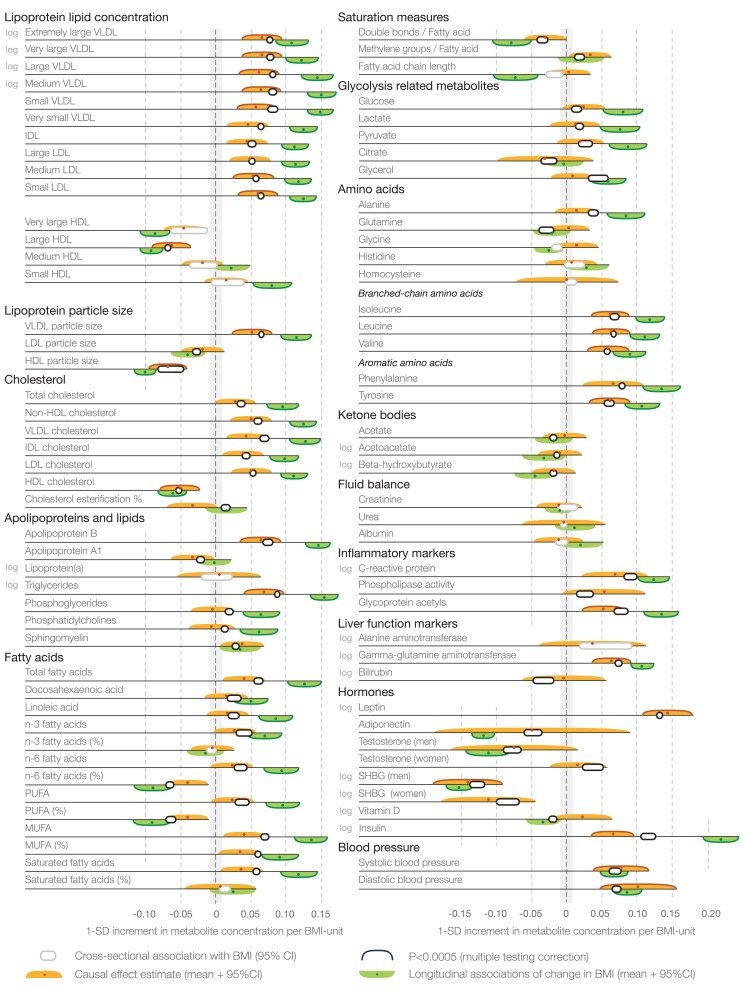
Cross-sectional associations, causal effect estimates, and longitudinal associations of BMI with systemic metabolites. Association magnitudes are in units of 1-SD metabolite concentration per 1-kg/m^2^ increment in BMI (cross-sectional associations [white] and causal effect estimates [orange]; *n* = 12,664), and change in metabolite concentration per 1-kg/m^2^ change in BMI at 6-y follow-up (longitudinal associations [green]; *n* = 1,488). Associations were adjusted for age and sex, and meta-analyzed for the four cohorts of adolescents and young adults. Colored dots indicate β-regression coefficients, colored shading denotes 95% confidence intervals, and boundaries on the shading indicate *p*<0.0005. All association magnitudes in absolute concentration units and exact *p*-values are listed in Table S3. MUFA, monounsaturated fatty acid; PUFA, polyunsaturated fatty acid; SHBG, sex hormone–binding globulin.

**Figure 5:**
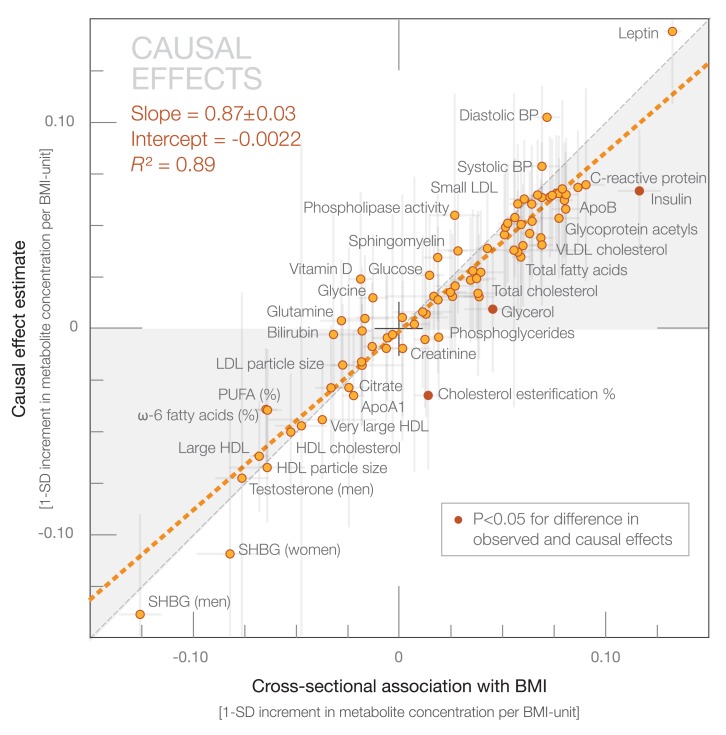
Correspondence between causal effect estimates and cross-sectional associations of BMI with systemic metabolites. Causal effect estimates based on Mendelian randomization are plotted against the metabolite associations with observed BMI based a cross-sectional study design. The orange dashed line denotes the linear fit of the correspondence. Darker dots indicate statistical differences between causal effect estimates and cross-sectional association magnitudes. The gray shaded areas serve to guide the eye for the slope of correspondence. BP, blood pressure; PUFA, polyunsaturated fatty acid; SHBG, sex hormone–binding globulin.

Causal effect estimates of BMI on the 82 metabolic measures are shown by the orange bars in Figure 4. The corresponding cross-sectional associations are indicated by white bars in Figure 4. The causal estimates based on Mendelian randomization gave rise to a metabolite association pattern highly concordant with that observed cross-sectionally: the effects of a 1-kg/m^2^ increment in BMI due to genetic predisposition closely matched the metabolic aberrations observed per 1-unit increment in observed BMI. The overall correspondence between the causal effect estimates and cross-sectional associations followed a straight line (*R* ^2^ = 0.89 [95% CI 0.84–0.93]; Figure 5), with a slope 0.87±0.03, consistent with causal effects of adiposity across the systemic metabolite profile. Cross-sectional association magnitudes and causal effect estimates did not significantly differ (*p*>0.0005) for any of the metabolic measures analyzed. The cross-sectional associations and causal effect estimates of each metabolite in absolute concentration units and with exact *p*-values are listed in Table S3.

In terms of individual metabolic risk factors, the causal estimates were significant for 24 of the metabolic measures at *p*<0.0005 (multiple testing corrected), and for a further 19 metabolic measures at *p*<0.05 (nominal significance). The strongest causal effect estimates were observed for VLDL and LDL lipids, branched-chain and aromatic amino acids, inflammatory markers, leptin, insulin, and blood pressure. Prominent inverse causal effects were found for large HDL lipid concentration, HDL particle size, and sex hormone–binding globulin.

Causal effect estimates were similar when using an unweighted gene score as the instrument for Mendelian randomization analyses (slope 0.82±0.04; *R* ^2^ = 0.85 [95% CI 0.79–0.91]), as well as when omitting the widely studied *FTO* locus (slope 0.79±0.04; *R* ^2^ = 0.82 [95% CI 0.75–0.89]) or any other individual variant from the gene score (Table 2). The causal effect estimates were also unaltered when adiposity was assessed by waist circumference utilizing the same genetic instrument (slope 0.87±0.04; *R* ^2^ = 0.86 [95% CI 0.81–0.91]). None of the metabolites were associated with the gene score when the model was adjusted for observed BMI (Figure S3), thus further arguing against pleiotropic effects of the genetic instrument. Associations for the small number of metabolites that displayed suggestive interaction by sex (*p*<0.05 for interaction) for the causal estimates are listed in Table S4.

### Weight Change and Metabolic Response

**Figure 6:**
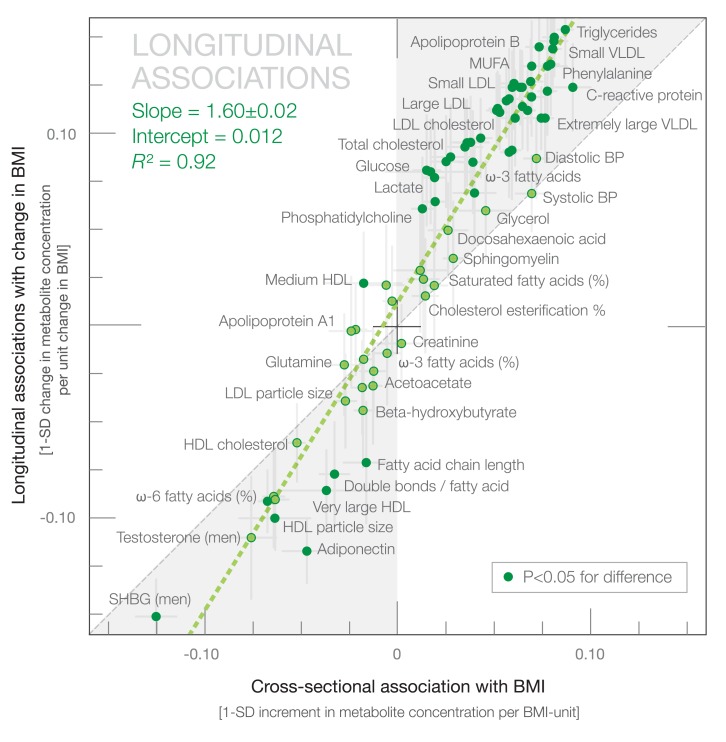
Correspondence between longitudinal associations of 6-y change in BMI with change in metabolites and cross-sectional associations. The green dashed line denotes the linear fit between longitudinal and cross-sectional observations. Darker dots indicate statistical differences between longitudinal and cross-sectional association magnitudes. The gray shaded areas serve to guide the eye for the slope of correspondence. BP, blood pressure; MUFA, monounsaturated fatty acid; SHBG, sex hormone–binding globulin.

To study the response of the metabolite profile to weight change, we examined associations between change in BMI and change in metabolite levels among 1,488 young adults at 6-y follow-up. These longitudinal associations are illustrated in Figure 4 by green bars. The concentration changes in 57 out of 76 metabolic measures were associated with 6-y change in BMI at *p*<0.0005. The metabolite changes followed an association pattern similar to the one observed in the cross-sectional analyses: those metabolites most strongly associated with BMI at one time point also displayed the highest responsiveness to changes in BMI over the follow-up period. However, the magnitudes of longitudinal associations were generally larger than the corresponding cross-sectional associations. The overall correspondence between longitudinal and cross-sectional associations followed a straight line (*R* ^2^ = 0.92 [95% CI 0.89–0.95]) with a slope of 1.60±0.05 (Figure 6).

Larger metabolic changes than expected based on the cross-sectional associations were observed for numerous lipoprotein lipid and cholesterol measures, fatty acids, and branched-chain amino acids, as well as inflammatory markers, adiponectin, and insulin. The magnitudes of the longitudinal associations in absolute concentration units are listed in Table S3. Similar results were obtained when the longitudinal analyses were further adjusted for baseline metabolite concentration, change in smoking status, change in alcohol intake, and change in physical activity (Figure S4). Similar results were also obtained when the longitudinal associations were examined at 10-y follow-up for the same study population (Figure S4; slope 1.60±0.06; *R* ^2^ = 0.91 [95% CI 0.87–0.95]). The longitudinal associations further replicated in the Pieksämäki cohort of 456 middle-aged adults with 6-y follow-up (Figure S4; slope 1.58±0.11; *R* ^2^ = 0.79 [95% CI 0.71–0.87]).

**Figure 7:**
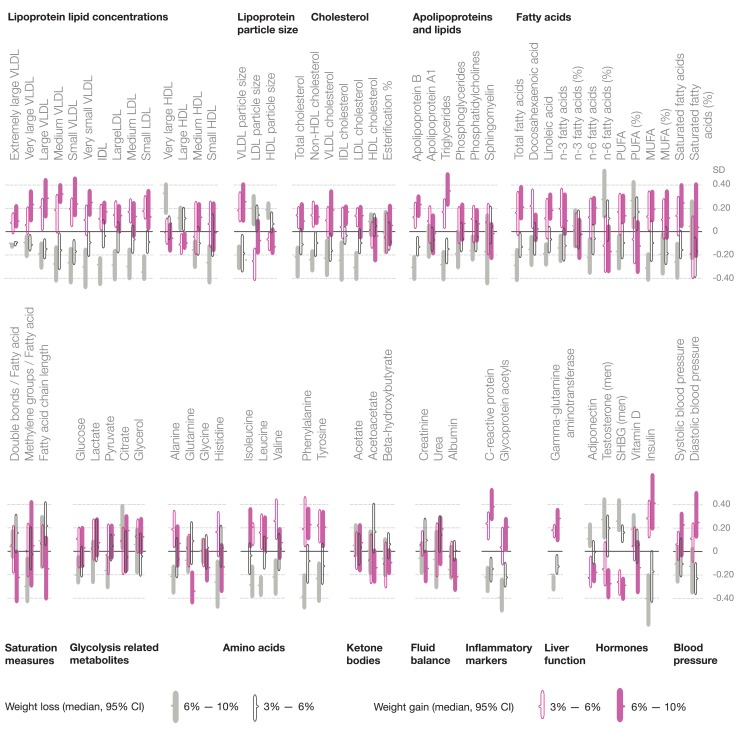
Metabolite changes paralleled by weight loss and weight gain. Median changes in metabolite concentrations at 6-y follow-up in four categories of weight change: filled gray bars, 6%–10% weight loss (mean [SD] loss 5.5±1.1 kg, *n* = 169); open black bars, 3%–6% weight loss (3.2±0.9 kg, *n* = 205); open purple bars, 3%–6% weight gain (3.2±0.9 kg, *n* = 168); filled purple bars, 6%–10% weight gain (5.9±1.7 kg, *n* = 138). The length of the bars indicates 95% confidence intervals of the median. The changes in metabolite concentrations are indicated in units of 1-SD baseline metabolite levels; metabobolite changes in absolute concentration units are listed in Table S5. MUFA, monounsaturated fatty acid; PUFA, polyunsaturated fatty acid; SHBG, sex hormone–binding globulin.

Changes in the metabolite profile with weight loss and weight gain at 6-y follow-up are illustrated in Figure 7. The metabolite changes are shown in SD units to ease comparison across metabolites; the corresponding metabolite changes in absolute concentration units are listed in Table S5. Widespread changes across the systemic metabolite profile were observed for both weight loss and weight gain in a graded manner. The metabolic changes paralleled by weight loss essentially mirrored the effects of weight gain: a weight loss of 6%–10% (mean 5.5 kg) was accompanied by lower levels of multiple cardiometabolic risk factors, including the lipoprotein subclass profile and diabetes-predictive amino acids, whereas a weight gain of 6%–10% (mean 5.9 kg) was associated with substantial metabolic changes in multiple pathways linked with higher cardiometabolic risk [31],[36]–[38]. Although HDL cholesterol was essentially unaltered for all weight change categories, substantial changes were observed within the HDL subclasses. Weight loss was also paralleled by decreased concentrations of monounsaturated fatty acids, improved ratio of polyunsaturated fatty acids to total fatty acids, and lower levels of chronic inflammation markers.

## Discussion

In this study of 12,664 healthy adolescents and young adults, elevated BMI was associated with adverse effects on numerous known and novel risk markers for cardiovascular disease and type 2 diabetes throughout the systemic metabolite profile [28],[31],[36]–[38]. Causal effect estimates obtained using Mendelian randomization followed a strikingly similar signature of metabolic aberrations. This strongly supports causative effects of adiposity across multiple cardiometabolic risk factors, even in young adults within the non-obese weight range. Causal metabolic effects of excess body weight included an unfavorable lipoprotein subclass profile and increased concentrations of branched-chain amino acids and inflammatory markers, as well as perturbed hormone levels and elevated blood pressure. These aberrations illustrate the diverse impact of adiposity on systemic metabolism, and demonstrate causal underpinnings for the clustering of metabolic risk factors commonly observed alongside obesity [39]. Consistent with the causative effect of adiposity on the metabolite profile, pronounced metabolic changes accompanied weight change at 6-y follow-up. Thus, despite genetic influences on both adiposity and the metabolite profile [29],[34], the results suggest that the metabolite profile is broadly modifiable in young adults through lifestyle changes.

The causal effects of adiposity across multiple metabolic measures corroborate prior Mendelian randomization studies, which have examined the role of the *FTO* locus on standard metabolic risk factors and cardiovascular disease [7],[14],[16],[40],[41]. The causal relationships are here extended to include lipoprotein subclass profiles and detailed lipid measures, branched-chain and aromatic amino acids, inflammation-linked glycoprotein acetyls [31], leptin, and sex hormone–binding globulin (Figure 4). The high consistency between the patterns of cross-sectional associations and casual effect estimates indicates that only little residual confounding contributes to the metabolic signatures of adiposity in early adulthood (Figure 5). The perturbed lipoprotein subclass pattern substantiates the causal role of adiposity in raising triglyceride-rich VLDL lipids and lowering HDL cholesterol [7],[14],[15], and further highlights the heterogeneity of HDL particles. Although high LDL cholesterol is conventionally not considered part of the dyslipidemic pattern of obesity [39], our results also indicate causative effects of elevated BMI on medium-sized and small LDL lipids. Branched-chain and aromatic amino acids are associated with the risk for cardiovascular disease and type 2 diabetes [37],[38]; their elevation due to higher adiposity could at least partly explain how these amino acids reflect the risk for future cardiometabolic disease. Causal effects on higher glycoprotein acetyl levels, which have recently been linked with the risk for both vascular and nonvascular mortality [31], suggest that increased adiposity contributes to this marker of chronic inflammation. The causality of these novel biomarkers in relation to disease outcomes still remains unknown; however, the causal role of adiposity across numerous metabolic risk markers could potentially contribute to the excess cardiovascular risk mediated by high BMI beyond the effects on raised blood pressure, cholesterol, and glucose [3].

Despite the heritable component of adiposity, BMI is a modifiable risk factor. Changes in BMI were paralleled by changes throughout the metabolite profile (Figures 4 and 7), which is consistent with the causal metabolic effects of adiposity. A systemic metabolite profile linked with high cardiometabolic risk in early adulthood is therefore not fixed once established, but can be reversed. These observational results are in line with weight loss interventions showing improved metabolic risk factors among overweight and obese individuals [15]–[20],[42]; the detailed metabolic profiling applied in this study extends the results to a more fine-grained molecular signature. The metabolite concentration changes were greater than anticipated—on average 60%—if effects were mediated directly through change in BMI rather than via more particular aspects of adiposity (Figure 6). This unexpectedly large metabolic response could possibly arise from concurrent lifestyle changes contributing to the obtained weight change, such as changes in diet or physical activity that are known to affect the metabolite profile [17],[26],[30]. The metabolic changes with weight loss and weight gain followed a graded trend, with adverse metabolic effects accompanying even a modest weight increase in this study population of largely non-obese individuals (Figure 7). These results are consistent with the continuous character of the metabolite associations with BMI observed cross-sectionally (Figure S2) and with the causal effects of adiposity across the comprehensive metabolite profile. The present study thus suggests unfavorable metabolic effects for any increase in BMI, without evidence of a threshold below which an increase in BMI would not affect the metabolite profile. Even though the individual metabolite deviations caused by a 1-kg/m^2^ increment in BMI were modest, the combined effects across the metabolite profile may have considerable implications. With the increasing trends in BMI worldwide, the adverse metabolic effects of adiposity observed in adolescents and young adults starting within the lean range of BMI may translate into direct consequences for cardiometabolic risk in the general population [1]–[3],[6],[36]–[38].

Our study has both strengths and limitations. BMI is a heterogeneous marker of adiposity; however, it predicts the risk of related complications and is relevant for large population studies [1]–[3],[6]. Pleiotropy is a concern in Mendelian randomization; the use of a multigenic instrument is helpful in this regard as it dilutes the effects of single genetic variant pleiotropy [11]–[13],[41]. Results were consistent when each individual variant was omitted in turn from the gene score (Table 2), suggesting that the metabolic effects are not attributable to a specific genetic variant. As far as we are aware, the multigenic score is a valid instrument; however, we acknowledge that the causal inference conducted depends on this assumption. Although observational associations and causal effect estimates matched across the metabolic measures analyzed, the inference of causality for certain metabolites and potential sex differences warrant stronger statistical power. The molecular coverage afforded by other metabolomics methods complementary to NMR may provide additional insights into the comprehensive metabolic effects of adiposity [36],[43]. Strengths of the study include detailed profiling across multiple metabolic pathways in large cohorts of healthy young adults and adolescents to quantify causal estimates and effects of weight change beyond established risk factors [28],[31],[36],[37].

The ideal body weight that healthy adults should strive to attain remains controversial [6],[44],[45]. The present study suggests widespread adverse metabolic effects with any increase in BMI among young adults within the non-obese weight range. However, modest weight loss was accompanied by multiple favorable changes in the systemic metabolite profile. The causative effect of adiposity on multiple cardiometabolic risk markers across the metabolite profile highlights the importance of population-level weight reduction as a key target for comprehensive risk factor control among young adults.

## Supporting Information

Figure S1 Correlations of the assayed metabolic measures.
(PDF)

Figure S2 Metabolite concentrations as a function of BMI on a continuous scale in young women and men.
(PDF)

Figure S3 Correspondence between gene score associations and cross-sectional associations of metabolic measures when the gene score associations are adjusted for observed BMI.
(PDF)

Figure S4 Associations of metabolite changes with change in BMI at 6-y and 10-y follow-up in the Cardiovascular Risk in Young Finns Study, and at 6-y follow-up in the Pieksämäki Study.
(PDF)

Table S1 Mean (SD) metabolite concentrations in each cohort and conversion factors to absolute units.
(PDF)

Table S2 Correlations between the gene score for elevated BMI and potential confounders.
(PDF)

Table S3 Cross-sectional associations, causal effect estimates, and longitudinal associations of BMI with systemic metabolites in absolute concentration units.
(PDF)

Table S4 Suggestive sex interactions in causal effect estimates of BMI on metabolites.
(PDF)

Table S5 Metabolite changes paralleled by weight loss and weight gain during 6-y follow-up in absolute concentration units.
(PDF)

Text S1 Study populations.
(PDF)

## Abbreviations

BMI: body mass index
HDL: high-density lipoprotein
LDL: low-density lipoprotein
NFBC66: Northern Finland Birth Cohort of 1966
NFBC86: Northern Finland Birth Cohort of 1986
NMR: nuclear magnetic resonance
SD: standard deviation
VLDL: very-low-density lipoprotein
YFS: Cardiovascular Risk in Young Finns Study
