# Supplementary material for: Metabolic Signatures of Adiposity in Young Adults: Mendelian Randomization Analysis and Effects of Weight Change
Source: PLoS Med. 2014 Dec 9;11(12):e1001765. doi: 10.1371/journal.pmed.1001765 (PMC4260795; doi:10.1371/journal.pmed.1001765)
Supplement: Figure S1 — Correlations of the assayed metabolic measures. (PDF) [file pmed.1001765.s001.pdf]

Figure S1: Correlations of the assayed metabolic measures.

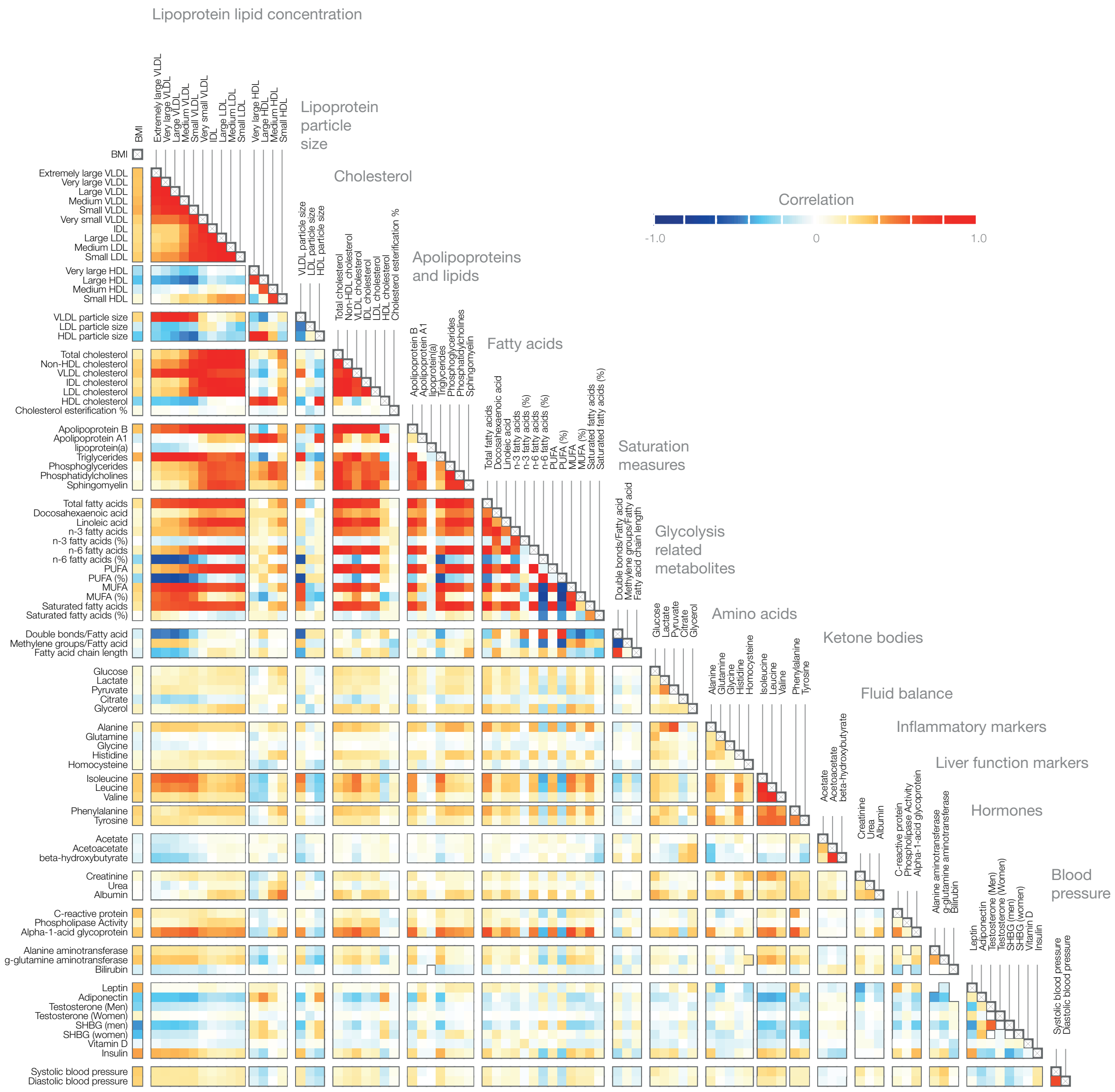

The color coding indicates Spearman’s correlation coefficients analyzed across the four study populations using inverse-variance weighted meta-analysis (n=12,664).
