# Supplementary material for: Metabolic Signatures of Adiposity in Young Adults: Mendelian Randomization Analysis and Effects of Weight Change
Source: PLoS Med. 2014 Dec 9;11(12):e1001765. doi: 10.1371/journal.pmed.1001765 (PMC4260795; doi:10.1371/journal.pmed.1001765)
Supplement: Figure S2 — Metabolite concentrations as a function of BMI on a continuous scale in young women and men. (PDF) [file pmed.1001765.s002.pdf]

Figure S2: Metabolite concentrations as a function of BMI on a continuous scale in young women and men.

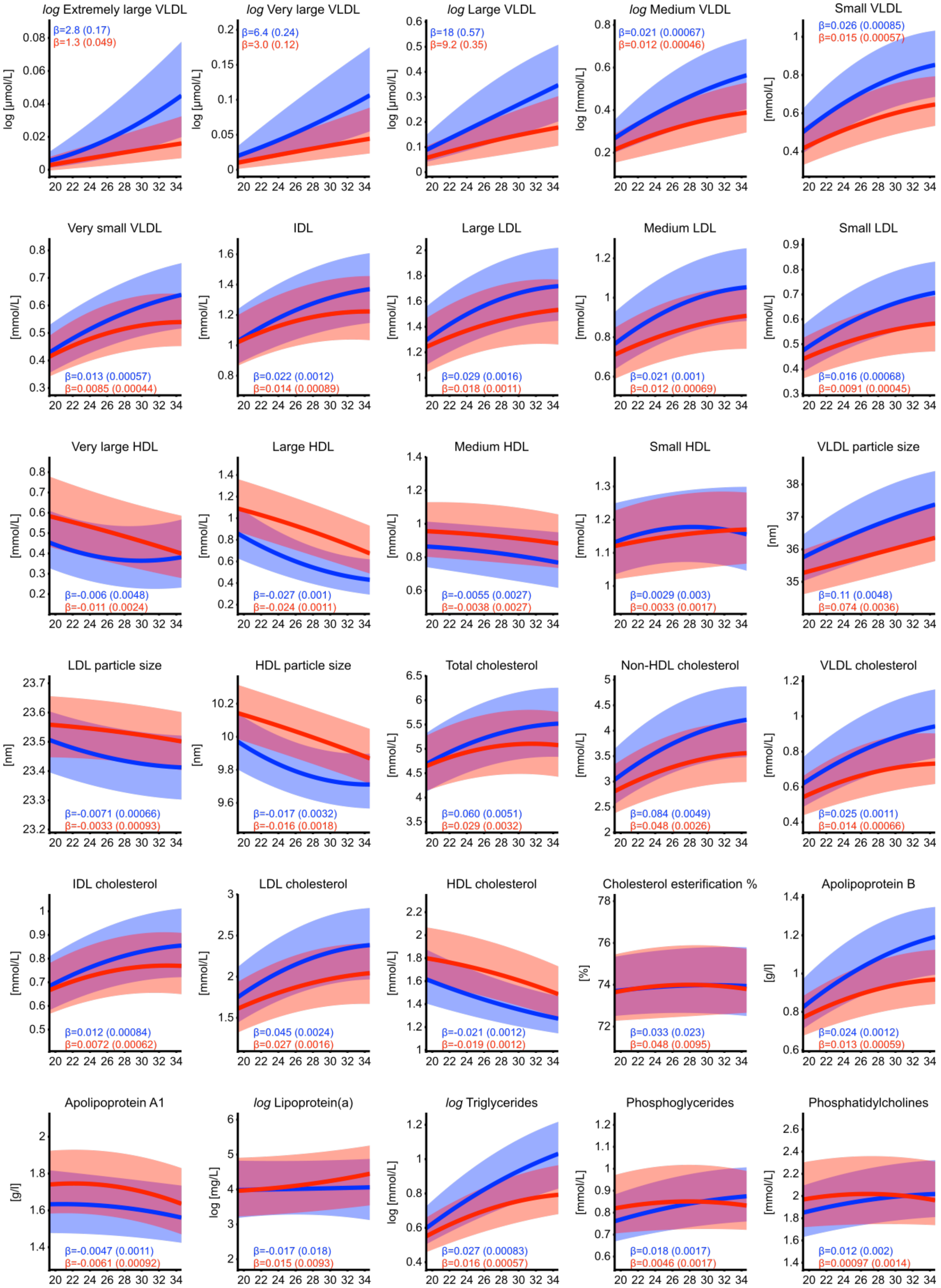

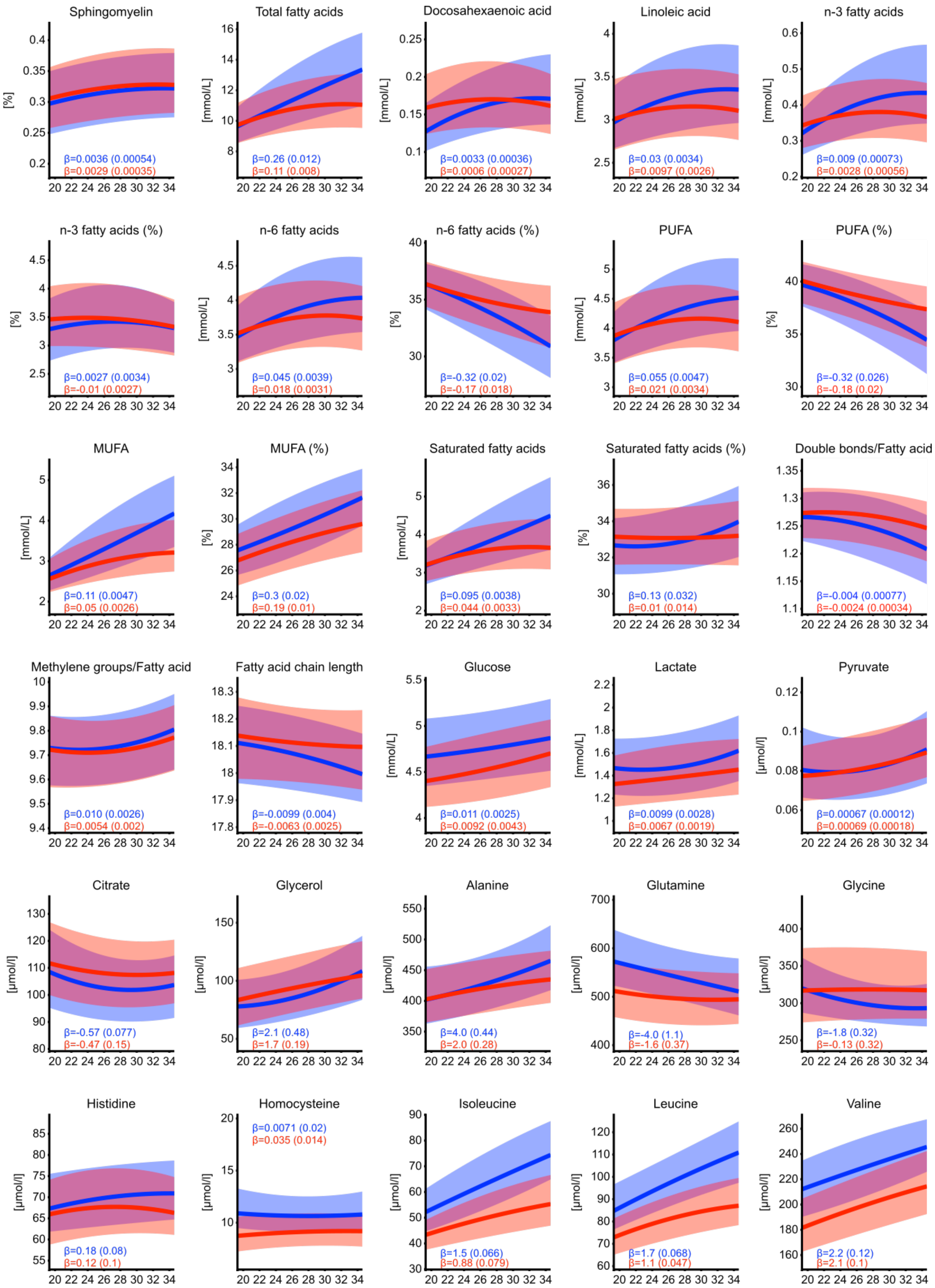

Sphingomyelin
[%]
β=0.0036 (0.00054)
β=0.0029 (0.00035)
Total fatty acids
[mmol/L]
β=0.26 (0.012)
β=0.11 (0.008)
Docosahexaenoic acid
[mmol/L]
β=0.0033 (0.00036)
β=0.0006 (0.00027)
Linoleic acid
[mmol/L]
β=0.03 (0.0034)
β=0.0097 (0.0026)
n-3 fatty acids
[mmol/L]
β=0.009 (0.00073)
β=0.0028 (0.00056)
n-3 fatty acids (%)
[%]
β=0.0027 (0.0034)
β=-0.01 (0.0027)
n-6 fatty acids
[mmol/L]
β=0.045 (0.0039)
β=0.018 (0.0031)
n-6 fatty acids (%)
[%]
β=-0.32 (0.02)
β=-0.17 (0.018)
PUFA
[mmol/L]
β=0.055 (0.0047)
β=0.021 (0.0034)
PUFA (%)
[%]
β=-0.32 (0.026)
β=-0.18 (0.02)
MUFA
[mmol/L]
β=0.11 (0.0047)
β=0.05 (0.0026)
MUFA (%)
[%]
β=0.3 (0.02)
β=0.19 (0.01)
Saturated fatty acids
[mmol/L]
β=0.095 (0.0038)
β=0.044 (0.0033)
Saturated fatty acids (%)
[%]
β=0.13 (0.032)
β=0.01 (0.014)
Double bonds/Fatty acid
β=-0.004 (0.00077)
β=-0.0024 (0.00034)
Methylene groups/Fatty acid
β=0.010 (0.0026)
β=0.0054 (0.002)
Fatty acid chain length
β=-0.0099 (0.004)
β=-0.0063 (0.0025)
Glucose
[mmol/L]
β=0.011 (0.0025)
β=0.0092 (0.0043)
Lactate
[mmol/L]
β=0.0099 (0.0028)
β=0.0067 (0.0019)
Pyruvate
[μmol/l]
β=0.00067 (0.00012)
β=0.00069 (0.00018)
Citrate
[μmol/l]
β=-0.57 (0.077)
β=-0.47 (0.15)
Glycerol
[μmol/l]
β=2.1 (0.48)
β=1.7 (0.19)
Alanine
[μmol/l]
β=4.0 (0.44)
β=2.0 (0.28)
Glutamine
[μmol/l]
β=-4.0 (1.1)
β=-1.6 (0.37)
Glycine
[μmol/l]
β=-1.8 (0.32)
β=-0.13 (0.32)
Histidine
[μmol/l]
β=0.18 (0.08)
β=0.12 (0.1)
Homocysteine
[μmol/l]
β=0.0071 (0.02)
β=0.035 (0.014)
Isoleucine
[μmol/l]
β=1.5 (0.066)
β=0.88 (0.079)
Leucine
[μmol/l]
β=1.7 (0.068)
β=1.1 (0.047)
Valine
[μmol/l]
β=2.2 (0.12)
β=2.1 (0.1)
20 22 24 26 28 30 32 34

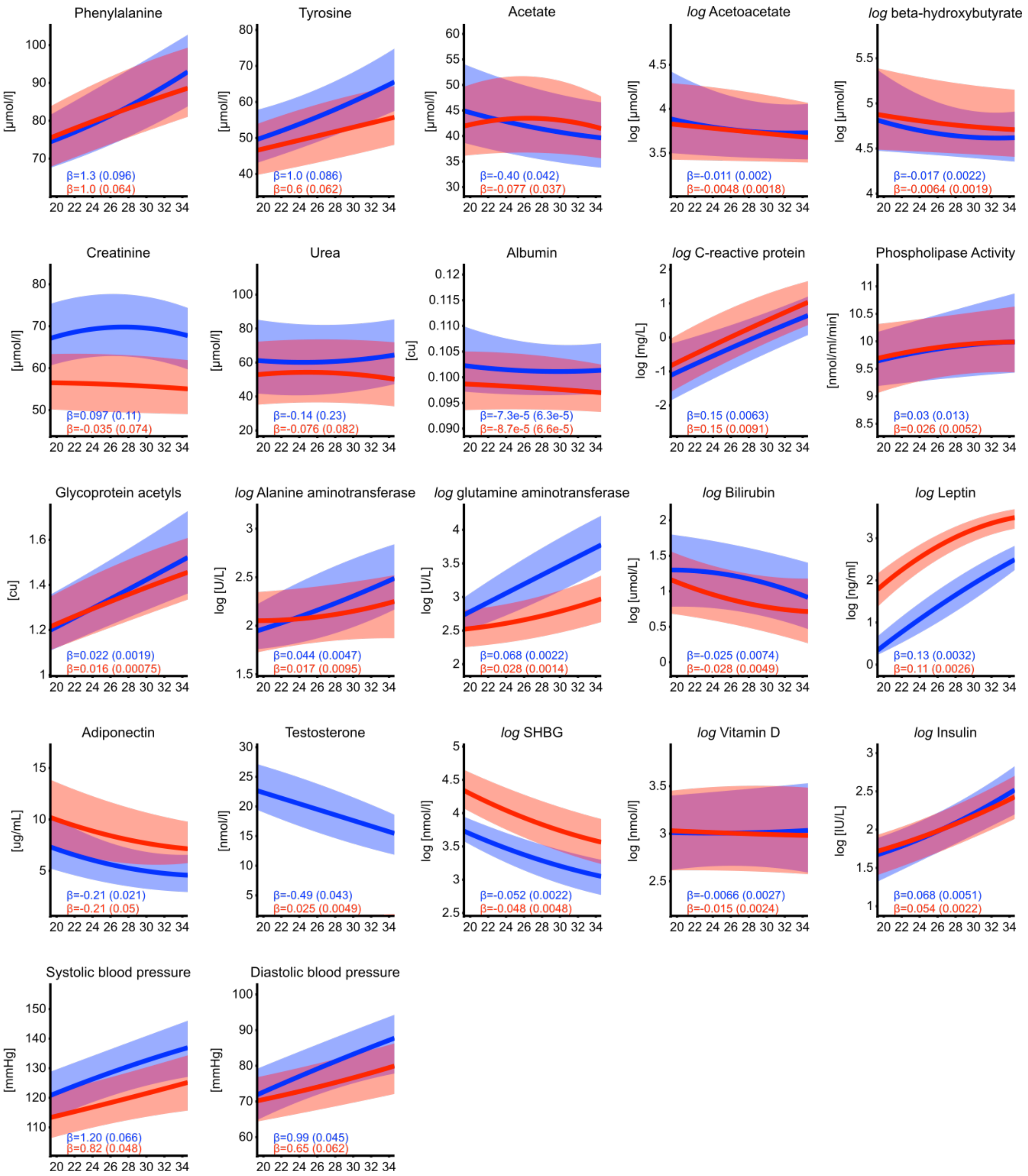

Characteristics of the metabolite concentrations as a function of BMI for young women (red; n=4,471) and men (blue; n=4,217). The thick lines indicate median and shaded areas indicate interquartile range of the metabolite concentrations fitted for each percentile of BMI with quadratic curve fits. The three cohorts of young adults were pooled for these descriptive analyses. Adolescents were omitted from these analyses since their BMI distribution is markedly lower. Similar trends were observed within all individual cohorts. The beta-regression coefficients (standard errors) from the cross-sectional analyses for the full study population in Figure 2 are indicated in absolute concentration units for women and men per 1-kg/m^2 increment in BMI.
