# Supplementary material for: Metabolic Signatures of Adiposity in Young Adults: Mendelian Randomization Analysis and Effects of Weight Change
Source: PLoS Med. 2014 Dec 9;11(12):e1001765. doi: 10.1371/journal.pmed.1001765 (PMC4260795; doi:10.1371/journal.pmed.1001765)
Supplement: Figure S3 — Correspondence between gene score associations and cross-sectional associations of metabolic measures when the gene score associations are adjusted for observed BMI. (PDF) [file pmed.1001765.s003.pdf]

Figure S3. Correspondence between gene score and cross-sectional associations with metabolic measures when the gene score associations are adjusted for observed BMI.

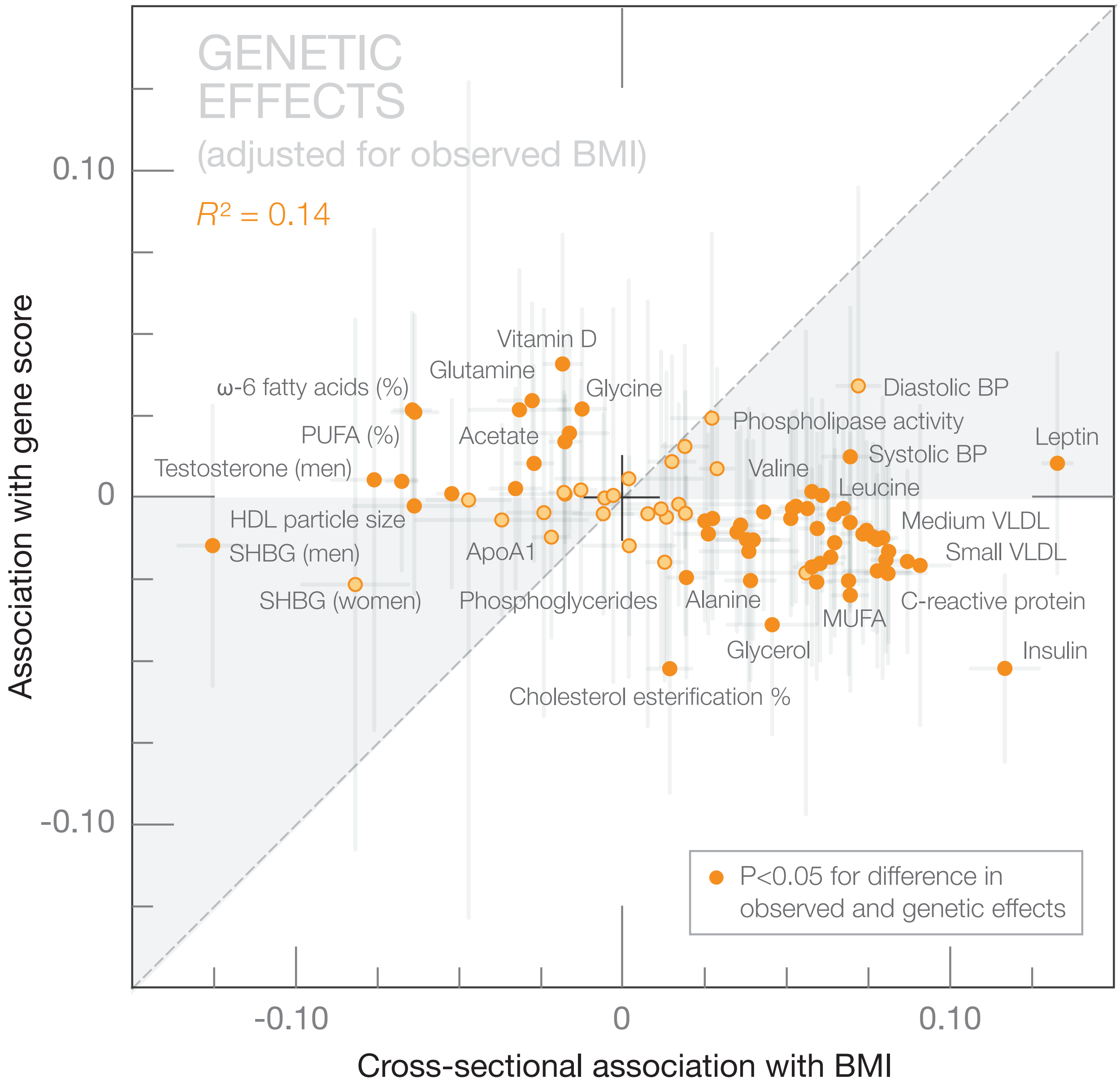

For the analyses of potential pleiotropy, the gene score for predisposition to elevated BMI was scaled to be associated with exactly 1-kg/m^2 unit increment in BMI; association magnitudes are hereby comparable to the magnitudes of the causal effect estimates shown in Figure 4 and Figure 5. Filled dots indicate significant difference between cross-sectional and genetic associations.

BP, blood pressure; MUFA, monounsaturated fatty acid; PUFA, polyunsaturated fatty acid; SHBG, sex hormone–binding globulin.
