# Supplementary material for: Metabolic Signatures of Adiposity in Young Adults: Mendelian Randomization Analysis and Effects of Weight Change
Source: PLoS Med. 2014 Dec 9;11(12):e1001765. doi: 10.1371/journal.pmed.1001765 (PMC4260795; doi:10.1371/journal.pmed.1001765)
Supplement: Figure S4 — Associations of metabolite changes with change in BMI at 6-y and 10-y follow-up in the Cardiovascular Risk in Young Finns Study, and at 6-y follow-up in the Pieksämäki Study. (PDF) [file pmed.1001765.s004.pdf]

Figure S4. Associations of metabolite changes with change in BMI at 6-year and 10-year follow-up in the Cardiovascular Risk in Young Finns Study, and at 6-year follow-up in the Pieksämäki Study.

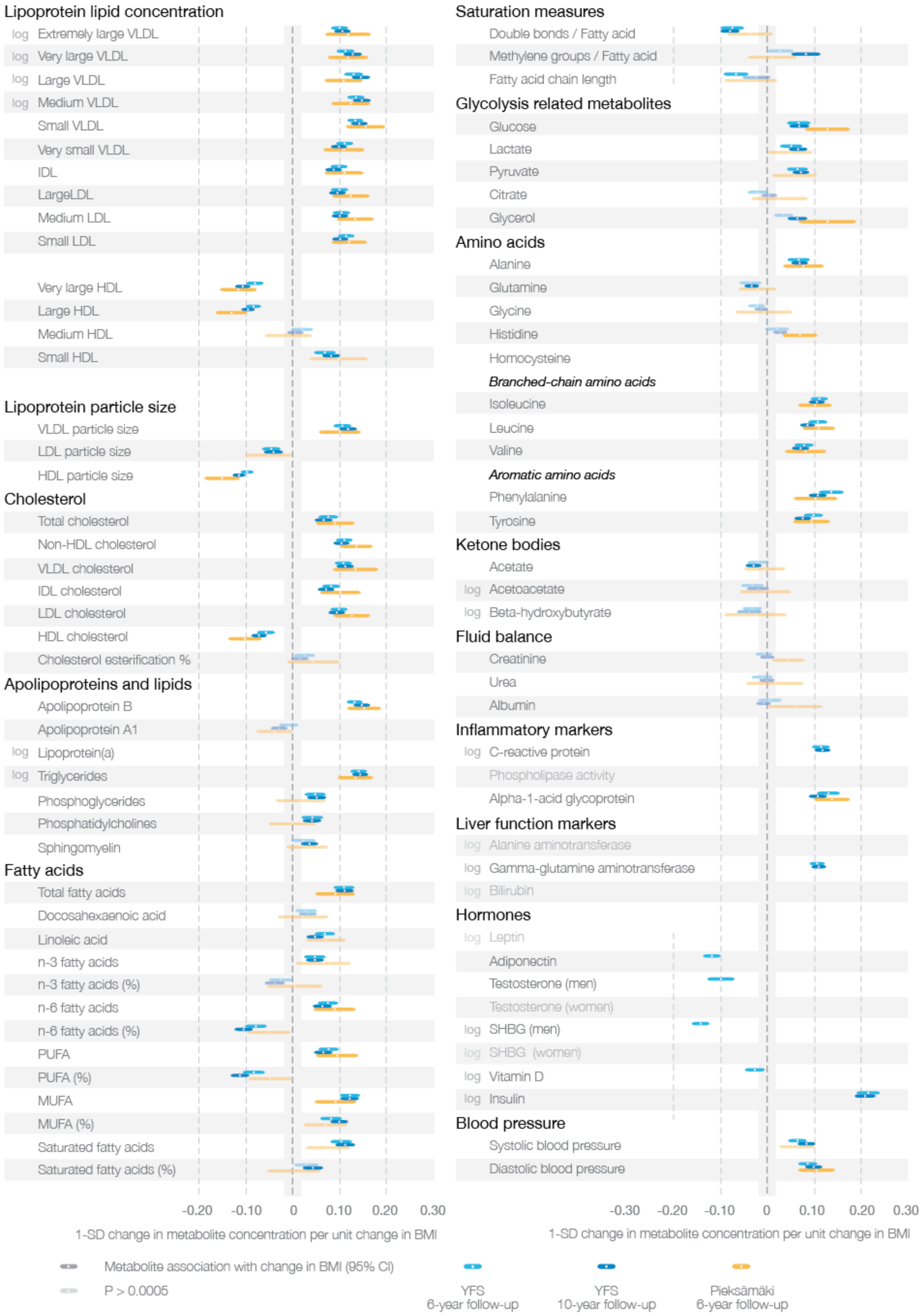

Association magnitudes are in units of change in metabolite concentration, scaled to 1-SD units at baseline, per 1-kg/m^2 change in BMI. White dots indicate β-regression coefficients, colored shading indicates 95% confidence intervals, and darker shading denotes $p < 0.0005$. Longitudinal associations were assessed in YFS during 6-year (n=1,488; light blue) and 10-year follow-up (n=1,372; dark blue). The longitudinal associations are also shown for 456 middle-aged persons from the Pieksämäki Study at 6-year follow-up (yellow). Associations were adjusted for sex and age, baseline metabolite concentration, change in smoking, change in alcohol intake, and change in physical activity index.
