# Supplementary material for: Metabolic Signatures of Adiposity in Young Adults: Mendelian Randomization Analysis and Effects of Weight Change
Source: PLoS Med. 2014 Dec 9;11(12):e1001765. doi: 10.1371/journal.pmed.1001765 (PMC4260795; doi:10.1371/journal.pmed.1001765)
Supplement: Table S1 — Mean (SD) metabolite concentrations in each cohort and conversion factors to absolute units. (PDF) [file pmed.1001765.s005.pdf]

Table S1: Mean metabolite concentrations in each cohort and conversion factors to absolute units.

Metabolite	NFBC 1986 (n=3,976)	NFBC 1966 (n=4,671)	Cardiovascular Risk in Young Finns (n=2,171)	FINRISK 1997 (n=1,846)	Conversion factor to absolute unit*
**Lipoprotein lipid concentration**					
Extremely large VLDL [μmol/L]	13 (19)	17 (27)	19 (29)	25 (29)	24
Very large VLDL [μmol/L]	47 (51)	52 (70)	56 (71)	49 (59)	56
Large VLDL [μmol/L]	187 (178)	212 (244)	251 (234)	148 (161)	150
Medium VLDL [mmol/L]	0.41 (0.25)	0.49 (0.35)	0.55 (0.32)	0.33 (0.22)	0.19
Small VLDL [mmol/L]	0.50 (0.18)	0.65 (0.27)	0.61 (0.23)	0.56 (0.22)	0.24
Very small VLDL [mmol/L]	0.41 (0.12)	0.52 (0.16)	0.51 (0.14)	0.54 (0.18)	0.16
IDL [mmol/L]	0.96 (0.25)	1.22 (0.35)	1.19 (0.29)	1.17 (0.33)	0.33
Large LDL [mmol/L]	1.16 (0.31)	1.54 (0.46)	1.48 (0.38)	1.39 (0.39)	0.43
Medium LDL [mmol/L]	0.67 (0.19)	0.92 (0.29)	0.88 (0.24)	0.81 (0.25)	0.27
Small LDL [mmol/L]	0.41 (0.13)	0.57 (0.19)	0.56 (0.16)	0.54 (0.18)	0.18
Very large HDL [mmol/L]	0.38 (0.19)	0.47 (0.22)	0.37 (0.21)	0.72 (0.25)	0.24
Large HDL [mmol/L]	0.76 (0.29)	0.85 (0.42)	0.79 (0.40)	0.93 (0.35)	0.37
Medium HDL [mmol/L]	0.85 (0.19)	0.93 (0.29)	1.00 (0.24)	0.74 (0.21)	0.26
Small HDL [mmol/L]	1.07 (0.14)	1.20 (0.20)	1.25 (0.16)	1.00 (0.13)	0.18
**Lipoprotein particle size**					
VLDL particle size [nm]	36.5 (1.30)	36.2 (1.37)	36.6 (1.37)	35.4 (1.12)	1.37
LDL particle size [nm]	23.6 (0.19)	23.5 (0.18)	23.5 (0.17)	23.5 (0.18)	0.18
HDL particle size [nm]	9.91 (0.22)	9.93 (0.26)	9.88 (0.26)	10.2 (0.25)	0.26
**Cholesterol**					
Total cholesterol [mmol/L]	4.20 (0.86)	5.32 (1.24)	5.00 (0.97)	4.98 (0.98)	1.15
Non-HDL cholesterol [mmol/L]	2.76 (0.75)	3.65 (1.12)	3.40 (0.92)	3.33 (0.97)	1.03
VLDL cholesterol [mmol/L]	0.61 (0.20)	0.77 (0.29)	0.68 (0.26)	0.72 (0.25)	0.26
IDL cholesterol [mmol/L]	0.63 (0.16)	0.80 (0.22)	0.74 (0.19)	0.75 (0.20)	0.21
LDL cholesterol [mmol/L]	1.52 (0.46)	2.09 (0.69)	1.98 (0.56)	1.86 (0.57)	0.63
HDL cholesterol [mmol/L]	1.45 (0.30)	1.67 (0.44)	1.60 (0.38)	1.64 (0.34)	0.39
Cholesterol esterification [%]	72.2 (1.1)	74.2 (2.1)	72.3 (1.7)	77.1 (3.0)	2.87
**Apolipoproteins and lipids**					
Apolipoprotein B [g/L]	0.79 (0.18)	0.98 (0.25)	0.95 (0.23)	0.92 (0.23)	0.24
Apolipoprotein A1 [g/L]	1.50 (0.22)	1.69 (0.25)	1.68 (0.24)	1.69 (0.23)	0.25
Lipoprotein(a) [mg/L]	—	—	143 (167)	95 (134)	1.16
Triglycerides [mmol/L]	0.95 (0.43)	1.16 (0.62)	1.28 (0.58)	1.05 (0.50)	0.23
Phosphoglycerides [mmol/L]	1.83 (0.37)	1.82 (0.50)	1.73 (0.39)	1.53 (0.39)	0.53
Phosphatidylcholines [mmol/L]	1.90 (0.31)	2.12 (0.52)	2.08 (0.43)	1.81 (0.39)	0.44
Sphingomyelin [mmol/L]	0.47 (0.08)	0.36 (0.08)	0.29 (0.06)	0.26 (0.06)	0.11
**Fatty acids**					
Total fatty acids [mmol/L]	11.0 (2.4)	11.5 (3.1)	10.9 (2.7)	11.1 (3.1)	2.85
Docosahexaenoic acid [mmol/L]	0.15 (0.05)	0.18 (0.07)	0.17 (0.07)	0.17 (0.07)	0.065
Linoleic acid [mmol/L]	2.74 (0.54)	3.34 (0.79)	3.12 (0.61)	3.21 (0.67)	0.72
n-3 fatty acids [mmol/L]	0.41 (0.11)	0.40 (0.15)	0.39 (0.14)	0.42 (0.14)	0.14
n-3 fatty acids/ total fatty acids [%]	3.8 (0.7)	3.5 (1.0)	3.6 (1.0)	3.8 (0.9)	0.89
n-6 fatty acids [mmol/L]	3.48 (0.64)	3.97 (0.93)	3.63 (0.70)	3.84 (0.83)	0.82
n-6 fatty acids/ total fatty acids [%]	31.8 (2.3)	34.9 (3.7)	33.9 (4.0)	35.1 (3.9)	3.68

PUFA [mmol/L]	3.89 (0.73)	4.37 (1.03)	4.01 (0.78)	4.27 (0.96)	0.92
PUFA/ total fatty acids [%]	35.5 (2.5)	38.3 (3.7)	37.4 (4.0)	38.9 (4.0)	3.72
MUFA [mmol/L]	2.67 (0.74)	3.28 (1.13)	3.32 (1.08)	3.12 (1.08)	1.05
MUFA/ total fatty acids [%]	24.1 (2.3)	28.2 (3.6)	30.2 (3.4)	27.9 (3.5)	3.93
Saturated fatty acids [mmol/L]	4.45 (0.95)	3.87 (1.16)	3.55 (1.00)	3.70 (1.12)	1.12
Saturated fatty acids/ total fatty acids [%]	40.4 (1.5)	33.5 (2.7)	32.4 (2.3)	33.2 (2.8)	4.12
**Saturation measures**					
Double bonds/ Fatty acid	1.15 (0.05)	1.26 (0.08)	1.27 (0.08)	1.25 (0.09)	0.091
Methylene groups/ Fatty acid	9.1 (0.3)	9.8 (0.2)	9.7 (0.2)	9.5 (0.3)	0.39
Fatty acid chain length	17.2 (0.2)	18.2 (0.2)	18.1 (0.2)	17.9 (0.3)	0.47
**Glycolysis related metabolites**					
Serum glucose [mmol/L]	4.54 (0.53)	4.81 (0.84)	4.92 (0.61)	4.3 (0.44)	0.69
Lactate [mmol/L]	1.47 (0.34)	1.57 (0.47)	1.56 (0.41)	1.23 (0.29)	0.42
Pyruvate [µmol/L]	74 (20)	89 (27)	86 (29)	72 (20)	0.025
Citrate [µmol/L]	103 (23)	106 (20)	112 (22)	107 (18)	21.2
Glycerol [µmol/L]	68 (28)	88 (36)	91 (40)	120 (49)	40.4
**Amino acids**					
Alanine [µmol/L]	406 (65)	439 (81)	424 (70)	414 (59)	72.7
Glutamine [µmol/L]	525 (72)	557 (102)	541 (94)	473 (67)	91.2
Glycine [µmol/L]	284 (48)	331 (73)	315 (72)	311 (58)	66.0
Histidine [µmol/L]	65 (10)	71 (14)	72 (12)	65 (10)	12.1
Homocysteine [µmol/l]	—	—	9.7 (2.9)	10.8 (3.1)	3.05
Isoleucine [µmol/L]	55 (13)	56 (17)	59 (17)	55 (18)	16.3
Leucine [µmol/L]	84 (16)	92 (21)	89 (21)	84 (20)	20.0
Valine [µmol/L]	203 (34)	214 (37)	213 (38)	197 (37)	37.0
Phenylalanine [µmol/L]	71 (11)	85 (15)	78 (12)	79 (12)	14.5
Tyrosine [µmol/L]	52 (11)	54 (13)	57 (12)	48 (13)	12.6
**Ketone bodies**					
Acetate [µmol/L]	46 (10)	43 (12)	44 (14)	49 (14)	12.1
Acetoacetate [µmol/L]	48 (36)	53 (36)	48 (31)	72 (43)	0.59
beta-hydroxybutyrate [µmol/L]	135 (101)	152 (106)	102 (74.3)	218 (150)	0.61
**Fluid balance**					
Creatinine [µmol/L]	55 (10)	65 (14)	66 (12)	56 (11)	13.0
Urea [µmol/L]	47 (23)	57 (28)	77 (34)	47 (24)	29.1
Albumin [cu]	0.10 (0.01)	0.10 (0.02)	0.10 (0.01)	0.10 (0.01)	0.014
**Inflammatory markers**					
C-reactive protein [mg/L]	0.9 (2.7)	1.9 (3.7)	1.7 (3.3)	1.7 (2.9)	1.62
Phospholipase activity [cu]	—	—	10.0 (1.0)	9.8 (1.0)	1.00
Glycoprotein acetyls [cu]	1.34 (0.21)	1.38 (0.27)	1.38 (0.26)	1.29 (0.21)	0.24
**Liver function markers**					
Alanine aminotransferase [U/L]	11.0 (6.7)	9.3 (4.6)	18.3 (12.5)	11.0 (6.7)	0.47
Gamma-glutamine aminotransferase [U/L]	14.6 (7.4)	21.2 (19.7)	25.0 (20.1)	25.6 (19.0)	0.59
Bilirubin [µmol/L]	4.0 (2.3)	3.8 (2.7)	—	—	0.81

**Hormones**					
Leptin [ng/mL]	—	—	10.7 (9.2)	9.7 (9.4)	0.90
Adiponectin [µg/mL]	—	—	9.4 (4.4)	6.4 (3.9)	4.46
Testosterone (Men) [nmol/L]	20.5 (6.6)	21.7 (6.4)	18.4 (5.4)	18.1 (6.6)	6.47
Testosterone (Women) [nmol/L]	1.7 (0.6)	1.1 (0.5)	1.6 (0.8)	1.2 (0.5)	0.65
SHBG (Men) [nmol/L]	32 (14)	34 (13)	31 (12)	—	0.41
SHBG (Women) [nmol/L]	67 (53)	64 (32)	83 (61)	—	0.58
Vitamin D [nmol/L]	—	20 (5.8)	44 (15)	13 (6.0)	0.62
Insulin [IU/L]	10.5 (5.0)	8.3 (3.7)	7.6 (5.0)	5.5 (3.9)	0.51
**Blood pressure**					
Systolic blood pressure [mmHg]	116 (13)	125 (13)	117 (13)	125 (14)	14.0
Diastolic blood pressure [mmHg]	68 (8)	77 (11)	71 (11)	77 (10)	10.9

* Multiplication factor (standard deviation of the metabolic measure in the study) to convert the association magnitudes reported in the figures to absolute concentrations units [e.g. mmol/L]. For example, each kg/m^2 unit higher BMI is associated with 0.0739 SD higher diastolic blood pressure, which corresponds to 0.806 mmHg higher diastolic blood pressure per BMI-unit (10.9 mmHg × 0.0739 SD). For metabolic measures that were log-transformed, the standard deviation of the transformed measure is indicated. Cross-sectional, instrumental variable and longitudinal association magnitudes in absolute concentration units are listed in Table S3.

Abbreviations: cu = standardized concentration unit; HDL, high-density lipoprotein; LDL, low-density lipoprotein; MUFA=monounsaturated fatty acids; PUFA=polyunsaturated fatty acids; SHBG=sex-hormone binding globulin; VLDL, very-low-density lipoprotein.
