# Supplementary material for: Metabolic Signatures of Adiposity in Young Adults: Mendelian Randomization Analysis and Effects of Weight Change
Source: PLoS Med. 2014 Dec 9;11(12):e1001765. doi: 10.1371/journal.pmed.1001765 (PMC4260795; doi:10.1371/journal.pmed.1001765)
Supplement: Table S2 — Correlations between the gene score for elevated BMI and potential confounders. (PDF) [file pmed.1001765.s006.pdf]

**Table S2: Correlations between the gene score for elevated BMI and potential confounders.**

| Potential confounder | NFBC 1986 | NFBC 1966 | Cardiovascular Risk in Young Finns | FINRISK 1997 |
|---|---|---|---|---|
| Sex | -0.025; P=0.11 | 0.014; P=0.32 | 0.019; P=0.38 | -0.014; P=0.54 |
| Age (years) | — | — | 0.0076; P=0.72 | 0.00059; P=0.98 |
| Smoking (yes/no) | -0.0073; P=0.65 | 0.0097; P=0.51 | -0.0057; P=0.79 | 0.04; P=0.082 |
| Alcohol usage (grams/week) | — | -0.023; P=0.12 | -0.025; P=0.25 | -0.027; P=0.25 |
| Physical activity index (Metabolic equivalent) | -0.0069; P=0.66 | -0.0025; P=0.86 | 0.016; P=0.46 | 0.034; P=0.14 (categorical index) |
| Socio-economic status (SES) | -0.03; P=0.05 (mother's SES) -0.02; P=0.16 (father's SES) | 0.01; P=0.48 (own SES) | 0.01; P=0.73 (own SES) | 0.03; P=0.15 (years of education) |

Correlations are Spearman's coefficients. Results were similar in sex-stratified analyses.
