# Supplementary material for: Metabolic Signatures of Adiposity in Young Adults: Mendelian Randomization Analysis and Effects of Weight Change
Source: PLoS Med. 2014 Dec 9;11(12):e1001765. doi: 10.1371/journal.pmed.1001765 (PMC4260795; doi:10.1371/journal.pmed.1001765)
Supplement: Table S3 — Cross-sectional associations, causal effect estimates, and longitudinal associations of BMI with systemic metabolites in absolute concentration units. (PDF) [file pmed.1001765.s007.pdf]

Table S3: Cross-sectional associations, causal effect estimates, and longitudinal associations of BMI with systemic metabolites in absolute concentration units.

Metabolite measure [unit]	*n*	Cross-sectional association [*beta* (95% *CI*); *P* per kg/m^2]	Causal effect estimate [*beta* (95% *CI*); *P* per kg/m^2]	*P* for diff. with cross-sectional association	Longitudinal association [*beta* (95% *CI*); *P* per kg/m^2 change]	*P* for diff. with sectional association
log Extremely large VLDL [µmol/L]	12,642	1.87 (1.77–1.97) P=9e-287	1.59 (0.902–2.28) P=6e-6	0.43	2.63 (2.08–3.19) P=8e-20	0.008
log Very large VLDL [µmol/L]	12,642	4.33 (4.07–4.59) P=2e-231	3.62 (2.06–5.19) P=5e-6	0.38	6.79 (5.53–8.06) P=7e-25	2e-4
log Large VLDL [µmol/L]	12,639	12.4 (11.7–13.0) P=4e-294	9.5 (5.21–13.8) P=1e-5	0.19	22.1 (18.7–25.6) P=2e-34	6e-8
log Medium VLDL [mmol/L]	12,643	0.0153 (0.0142–0.0164) P=5e-163	0.0121 (0.00693–0.0173) P=5e-6	0.24	0.0282 (0.0244–0.032) P=7e-45	2e-10
Small VLDL [mmol/L]	12,643	0.0194 (0.0177–0.0211) P=3e-114	0.0138 (0.00718–0.0203) P=4e-5	0.10	0.0354 (0.031–0.0398) P=1e-51	3e-11
Very small VLDL [mmol/L]	12,643	0.0102 (0.0095–0.0109) P=1e-188	0.00732 (0.00249–0.0121) P=0.003	0.25	0.0198 (0.0168–0.0229) P=2e-34	2e-9
IDL [mmol/L]	12,643	0.0169 (0.0152–0.0185) P=1e-90	0.0148 (0.00514–0.0245) P=0.003	0.68	0.0367 (0.0304–0.043) P=7e-29	2e-9
Large LDL [mmol/L]	12,643	0.0221 (0.0202–0.024) P=6e-120	0.0207 (0.00831–0.0332) P=0.001	0.83	0.0481 (0.0399–0.0563) P=1e-29	1e-9
Medium LDL [mmol/L]	12,643	0.0153 (0.0141–0.0164) P=4e-149	0.0144 (0.00664–0.0222) P=0.0003	0.84	0.0316 (0.0265–0.0367) P=4e-32	1e-9
Small LDL [mmol/L]	12,643	0.0118 (0.011–0.0125) P=2e-202	0.0109 (0.00572–0.0161) P=4e-5	0.75	0.0225 (0.0191–0.026) P=5e-36	2e-9
Very large HDL [mmol/L]	12,641	-0.00903 (-0.0154– -0.00266) P=0.005	-0.0109 (-0.0179– -0.00387) P=0.002	0.70	-0.0209 (-0.026– -0.0158) P=3e-15	0.005
Large HDL [mmol/L]	12,641	-0.0253 (-0.027– -0.0236) P=4e-185	-0.0234 (-0.0335– -0.0133) P=6e-6	0.71	-0.0341 (-0.0402– -0.0281) P=1e-27	0.006
Medium HDL [mmol/L]	12,643	-0.00455 (-0.00962–0.000526) P=0.08	-0.00471 (-0.0124–0.00295) P=0.22	0.97	0.00573 (-0.0011–0.0126) P=0.10	0.02
Small HDL [mmol/L]	12,643	0.00315 (-0.00115–0.00745) P=0.10	0.00279 (-0.00279–0.00837) P=0.33	0.92	0.0147 (0.00978–0.0197) P=6e-9	5e-4
VLDL particle size [nm]	12,643	0.0888 (0.0832–0.0945) P=4e-206	0.0707 (0.0319–0.11) P=0.0004	0.37	0.156 (0.126–0.186) P=1e-23	2e-5
LDL particle size [nm]	12,643	-0.00494 (-0.00596– -0.00392) P=2e-21	-0.0033 (-0.00872–0.00212) P=0.23	0.56	-0.00712 (-0.0116– -0.00268) P=0.002	0.35
HDL particle size [nm]	12,642	-0.0166 (-0.0214– -0.0118) P=1e-11	-0.0177 (-0.0246– -0.0107) P=6e-7	0.8	-0.026 (-0.0299– -0.0221) P=4e-37	3e-3
Total cholesterol [mmol/L]	12,643	0.0415 (0.0344–0.0486) P=3e-30	0.0318 (-0.0025–0.066) P=0.07	0.59	0.11 (0.0838–0.135) P=1e-16	6e-7
Non-HDL cholesterol [mmol/L]	12,643	0.0612 (0.0558–0.0667) P=3e-107	0.0515 (0.022–0.0811) P=0.0006	0.53	0.128 (0.108–0.147) P=2e-36	8e-11
VLDL cholesterol [mmol/L]	12,643	0.0181 (0.0165–0.0197)	0.0114 (0.00398–0.0189)	0.085	0.0333 (0.0276–0.039)	5e-7

		P=8e-110	P=0.003		P=6e-29	
IDL cholesterol [mmol/L]	12,643	0.0089 (0.00771–0.0101) P=7e-49	0.00794 (0.00183–0.014) P=0.01	0.76	0.0201 (0.0159–0.0243) P=2e-20	5e-7
LDL cholesterol [mmol/L]	12,643	0.0335 (0.0306–0.0364) P=1e-116	0.032 (0.0137–0.0503) P=0.0006	0.88	0.0701 (0.058–0.0822) P=8e-29	8e-9
HDL cholesterol [mmol/L]	12,642	-0.0202 (-0.0218– -0.0186) P=2e-133	-0.0196 (-0.0305– -0.0086) P=0.0005	0.91	-0.0235 (-0.0313– -0.0157) P=5e-9	0.42
Cholesterol esterification % [%]	12,511	0.0414 (0.0223–0.0605) P=2e-5	-0.0943 (-0.198–0.00916) P=0.07	0.011	0.0448 (-0.0402–0.13) P=0.3	0.94
Apolipoprotein B [g/l]	11,970	0.0175 (0.0159–0.0191) P=4e-100	0.0151 (0.00812–0.0221) P=2e-5	0.51	0.0346 (0.0304–0.0388) P=3e-54	8e-14
Apolipoprotein A1 [g/l]	11,970	-0.00546 (-0.00686– -0.00406) P=2e-14	-0.00826 (-0.016– -0.000503) P=0.04	0.49	-0.00046 (-0.0064–0.0055) P=0.90	0.11
log Lipoprotein(a) [mg/L]	3,567	0.00219 (-0.0236–0.028) P=0.9	0.00574 (-0.0635–0.075) P=0.87	0.92	—	—
log Triglycerides [mmol/L]	12,643	0.0204 (0.0195–0.0214) P<1e-300	0.016 (0.00947–0.0224) P=1e-6	0.18	0.0361 (0.0316–0.0406) P=1e-51	3e-11
Phosphoglycerides [mmol/L]	12,526	0.0104 (0.00742–0.0133) P=5e-12	-0.00246 (-0.0186–0.0137) P=0.77	0.13	0.0343 (0.0207–0.048) P=9e-7	8e-4
Phosphatidyl-cholines [mmol/L]	12,520	0.00571 (0.00333–0.00809) P=3e-6	-0.00253 (-0.0172–0.0121) P=0.74	0.28	0.027 (0.0152–0.0388) P=8e-6	5e-4
Sphingomyelin [mmol/L]	12,481	0.00311 (0.00258–0.00365) P=3e-30	0.00402 (0.000739–0.00729) P=0.02	0.59	0.00378 (0.000593–0.00698) P=0.02	0.68
Total fatty acids [mmol/L]	12,517	0.172 (0.154–0.191) P=2e-71	0.114 (0.0291–0.198) P=0.008	0.18	0.36 (0.292–0.427) P=2e-24	2e-7
Docosahexaenoic acid [mmol/L]	12,506	0.0017 (0.00105–0.00236) P=4e-7	0.000986 (-0.000991–0.00296) P=0.33	0.50	0.00323 (0.00164–0.00483) P=7e-5	0.08
Linoleic acid [mmol/L]	12,535	0.0181 (0.0125–0.0237) P=3e-10	0.0124 (-0.00945–0.0342) P=0.27	0.62	0.0614 (0.0443–0.0784) P=2e-12	2e-6
n-3 fatty acids [mmol/L]	12,542	0.00541 (0.00392–0.00689) P=9e-13	0.00364 (-0.000428–0.0077) P=0.08	0.42	0.00936 (0.00599–0.0127) P=6e-8	0.04
n-3 fatty acids [%]	12,528	-0.00487 (-0.0113–0.00152) P=0.10	-0.00441 (-0.0319–0.0231) P=0.75	0.97	-0.0127 (-0.0365–0.0111) P=0.3	0.53
n-6 fatty acids [mmol/L]	12,521	0.0288 (0.0213–0.0364) P=5e-14	0.0191 (-0.00591–0.044) P=0.14	0.46	0.0767 (0.0568–0.0966) P=6e-14	1e-5
n-6 fatty acids [%]	12,528	-0.237 (-0.257– -0.217) P=1e-117	-0.146 (-0.255– -0.0369) P=0.009	0.11	-0.326 (-0.424– -0.229) P=6e-11	0.08
PUFA [mmol/L]	12,519	0.0349 (0.0261–0.0437) P=6e-15	0.0219 (-0.00585–0.0497) P=0.12	0.38	0.0878 (0.066–0.11) P=8e-15	1e-5
PUFA [%]	12,519	-0.237 (-0.262– -0.211) P=1e-71	-0.149 (-0.259– -0.0385) P=0.008	0.13	-0.336 (-0.434– -0.238) P=2e-11	0.05
MUFA [mmol/L]	12,529	0.0731 (0.0675–0.0787) P=2e-145	0.0422 (0.0113–0.073) P=0.007	0.053	0.142 (0.117–0.166) P=1e-28	8e-8
MUFA [%]	12,512	0.233 (0.216–0.25)	0.135 (0.0179–0.252)	0.10	0.359 (0.259–0.459)	0.04

		P=2e-159	P=0.02		P=3e-12	
Saturated fatty acids [mmol/L]	12,500	0.0651 (0.0592–0.071) P=3e-102	0.0412 (0.00756–0.0749) P=0.02	0.17	0.133 (0.104–0.162) P=1e-18	8e-6
Saturated fatty acids [%]	12,512	0.0552 (0.0226–0.0878) P=0.0009	0.0271 (-0.179–0.234) P=0.78	0.79	0.10 (-0.0355–0.236) P=0.10	0.53
Double bonds/ Fatty acid	12,525	-0.00299 (-0.00369– -0.00228) P=9e-17	-0.00266 (-0.00543–0.00011) P=0.06	0.82	-0.00703 (-0.0095– -0.00456) P=3e-8	0.002
Methylene groups/ Fatty acid	12,519	0.00743 (0.00477–0.0101) P=5e-8	0.0133 (0.0013–0.0253) P=0.03	0.35	0.0082 (-0.00473–0.0211) P=0.2	0.91
Fatty acid chain length	12,489	-0.00767 (-0.0132– -0.00212) P=0.007	0.0021 (-0.0124–0.0166) P=0.78	0.22	-0.0335 (-0.0484– -0.0186) P=1e-5	0.001
Glucose [mmol/L]	12,630	0.0104 (0.00562–0.0152) P=2e-5	0.0175 (-0.00319–0.0382) P=0.10	0.51	0.0558 (0.0361–0.0755) P=4e-8	1e-5
Lactate [mmol/L]	12,629	0.00797 (0.0055–0.0104) P=3e-10	0.00561 (-0.00924–0.0205) P=0.46	0.76	0.032 (0.0203–0.0437) P=1e-7	8e-5
Pyruvate [µmol/l]	12,629	0.000701 (0.00046–0.00095) P=2e-8	0.000516 (-0.00035–0.00138) P=0.24	0.69	0.00224 (0.00155–0.00293) P=3e-10	4e-5
Citrate [µmol/l]	12,610	-0.511 (-0.747– -0.274) P=2e-5	-0.616 (-2.06–0.824) P=0.40	0.89	-0.0565 (-0.656–0.543) P=0.90	0.17
Glycerol [µmol/l]	12,550	1.85 (1.3–2.4) P=6e-11	0.364 (-0.873–1.6) P=0.56	0.03	2.42 (1.38–3.47) P=6e-6	0.34
Alanine [µmol/l]	12,632	2.84 (2.3–3.39) P=1e-24	1.09 (-1.11–3.3) P=0.33	0.13	6.19 (4.26–8.11) P=4e-10	0.001
Glutamine [µmol/l]	12,617	-2.52 (-3.45– -1.59) P=1e-7	0.309 (-2.41–3.03) P=0.82	0.05	-1.84 (-4.24–0.553) P=0.10	0.60
Glycine [µmol/l]	12,578	-0.818 (-1.28– -0.355) P=0.0005	0.957 (-1.07–2.99) P=0.36	0.09	-1.55 (-2.94– -0.167) P=0.03	0.33
Histidine [µmol/l]	12,624	0.143 (-0.0276–0.313) P=0.1	0.0927 (-0.356–0.542) P=0.69	0.84	0.351 (-0.037–0.739) P=0.08	0.34
Homocysteine [µmol/l]	3,729	0.0236 (0.00063–0.0465) P=0.04	0.00511 (-0.214–0.224) P=0.96	0.87	—	—
Isoleucine [µmol/l]	12,628	1.13 (1.03–1.24) P=2e-105	1.03 (0.594–1.47) P=4e-6	0.65	1.94 (1.61–2.27) P=2e-29	5e-6
Leucine [µmol/l]	12,616	1.35 (1.27–1.43) P=7e-249	1.29 (0.748–1.82) P=3e-6	0.82	2.24 (1.82–2.65) P=4e-25	4e-5
Valine [µmol/l]	12,093	2.14 (1.99–2.29) P=4e-164	2.22 (1.15–3.29) P=5e-5	0.89	3.34 (2.48–4.2) P=7e-14	0.007
Phenylalanine [µmol/l]	12,631	1.15 (1.09–1.21) P=2e-292	0.974 (0.35–1.60) P=0.002	0.58	1.97 (1.58–2.35) P=3e-23	4e-5
Tyrosine [µmol/l]	12,634	0.770 (0.678–0.861) P=1e-60	0.787 (0.423–1.15) P=2e-5	0.93	1.36 (1.05–1.67) P=2e-17	4e-4
Acetate [µmol/l]	12,611	-0.213 (-0.268– -0.159) P=2e-14	-0.0188 (-0.388–0.351) P=0.92	0.31	-0.209 (-0.534–0.116) P=0.2	0.98
log Acetoacetate [µmol/l]	12,525	-0.00751 (-0.0102– -0.00485)	-0.00538 (-0.0236–0.0128)	0.82	-0.0183 (-0.0361– -0.00054)	0.24

		P=3e-8	P=0.56		P=0.04	
log beta-hydroxy-butyrate [μmol/l]	12,510	-0.0109<br>(-0.0136– -0.00814)<br>P=5e-15	-0.01<br>(-0.0287–0.00866)<br>P=0.29	0.93	-0.0266<br>(-0.0446– -0.00867)<br>P=0.004	0.09
Creatinine [μmol/l]	12,595	0.0264<br>(-0.155–0.208)<br>P=0.80	-0.131<br>(-0.559–0.296)<br>P=0.55	0.51	-0.119<br>(-0.382–0.144)<br>P=0.4	0.37
Urea [μmol/l]	12,580	-0.0832<br>(-0.213–0.0467)<br>P=0.20	-0.0976<br>(-1.81–1.61)<br>P=0.91	0.99	0.376<br>(-0.452–1.2)<br>P=0.4	0.28
Albumin [cu]	12,643	-8.2e-5<br>(-2.1e-4–4.3e-5)<br>P=0.20	-0.00014<br>(-6.3e-4–3.5e-4)<br>P=0.58	0.82	0.000296<br>(-1.4e-4–7e-4)<br>P=0.2	0.10
log C-reactive protein [mg/L]	12,462	0.147<br>(0.133–0.162)<br>P=3e-84	0.112<br>(0.0364–0.188)<br>P=0.004	0.37	0.201<br>(0.164–0.237)<br>P=7e-26	0.008
Phospholipase activity [nmol/ml/min]	3,682	0.0273<br>(0.0151–0.0395)<br>P=1e-5	0.0545<br>(-0.00444–0.113)<br>P=0.07	0.38	—	—
Glycoprotein acetyls [cu]	12,636	0.0189<br>(0.0165–0.0213)<br>P=5e-53	0.0129<br>(0.00579–0.0199)<br>P=0.0004	0.12	0.0327<br>(0.0267–0.0387)<br>P=5e-26	3e-5
log Alanine amino-transferase [U/L]	6,996	0.0263<br>(0.00902–0.0437)<br>P=0.003	0.0177<br>(-0.0181–0.0535)<br>P=0.33	0.67	—	—
log gamma-glutamine amino-transferase [U/L]	12,156	0.0437<br>(0.0404–0.0469)<br>P=2e-152	0.0376<br>(0.0216–0.0536)<br>P=4e-6	0.46	0.0633<br>(0.0538–0.0727)<br>P=4e-37	1e-4
log Bilirubin [μmol/L]	8,067	-0.0256<br>(-0.0379– -0.0133)<br>P=4e-5	-0.00267<br>(-0.0504–0.045)<br>P=0.91	0.36	—	—
log Leptin [ng/ml]	3,761	0.12<br>(0.116–0.124)<br>P<1e-300	0.13<br>(0.0981–0.162)<br>P=1e-15	0.55	—	—
Adiponectin [ug/mL]	3,922	-0.21<br>(-0.266– -0.154)<br>P=2e-13	-0.212<br>(-0.833–0.408)<br>P=0.50	0.99	-0.522<br>(-0.596– -0.448)<br>P=3e-41	5e-11
Testosterone (Men) [nmol/l]	6,023	-0.491<br>(-0.575– -0.408)<br>P=2e-30	-0.472<br>(-1.06–0.111)<br>P=0.11	0.95	-0.712<br>(-0.917– -0.506)<br>P=2e-11	0.05
Testosterone (Women) [nmol/l]	6,206	0.0249<br>(0.0153–0.0345)<br>P=4e-7	0.0108<br>(-0.0151–0.0366)<br>P=0.41	0.31	—	—
log SHBG (men) [nmol/l]	5,239	-0.052<br>(-0.0563– -0.0476)<br>P=1e-120	-0.0576<br>(-0.0779– -0.0374)<br>P=2e-8	0.59	-0.0626<br>(-0.0706– -0.0546)<br>P=2e-45	0.02
log SHBG (women) [nmol/l]	4,550	-0.0476<br>(-0.0571– -0.0382)<br>P=6e-23	-0.0639<br>(-0.103– -0.0246)<br>P=0.001	0.43	—	—
log Vitamin D [nmol/l]	7,126	-0.0113<br>(-0.0148– -0.00784)<br>P=2e-10	0.0145<br>(-0.0115–0.0406)<br>P=0.27	0.05	-0.0197<br>(-0.0346– -0.00487)<br>P=0.01	0.28
log Insulin [IU/L]	12,262	0.0592<br>(0.0539–0.0645)<br>P=1e-106	0.0336<br>(0.0185–0.0487)<br>P=1e-5	0.002	0.111<br>(0.0982–0.124)<br>P=1e-59	2e-13
Systolic blood pressure [mmHg]	12,635	0.973<br>(0.857–1.09)<br>P=9e-61	1.09<br>(0.551–1.64)<br>P=8e-5	0.67	0.962<br>(0.688–1.24)<br>P=9e-12	0.94
Diastolic blood pressure [mmHg]	12,627	0.788<br>(0.717–0.859)<br>P=4e-106	1.12<br>(0.527–1.70)<br>P=0.0002	0.28	0.951<br>(0.726–1.18)<br>P=2e-16	0.17

Metabolite association magnitudes and causal effect estimates shown in Figure 3 are scaled to absolute concentrations units by multiplying by the SD for each metabolite measure (listed in Table S1). P-value for difference between cross-sectional association magnitudes and causal effect estimates, or longitudinal associations, were calculated using a *Z*-statistic. cu = standardized concentration unit.
