# Supplementary material for: Metabolic Signatures of Adiposity in Young Adults: Mendelian Randomization Analysis and Effects of Weight Change
Source: PLoS Med. 2014 Dec 9;11(12):e1001765. doi: 10.1371/journal.pmed.1001765 (PMC4260795; doi:10.1371/journal.pmed.1001765)
Supplement: Table S4 — Suggestive sex interactions in causal effect estimates of BMI on metabolites. (PDF) [file pmed.1001765.s008.pdf]

Table S4: Suggestive sex interactions in causal effect effect estimates of BMI on metabolites.

Metabolite measure [unit]	*P* for genetic interaction	Sex	*n*	Cross-sectional association [beta (95%CI); P per kg/m^2]	Causal effect estimate [beta (95%CI); P per kg/m^2]	*P*-value for difference*
log Extremely large VLDL [μmol/L]	0.0006	Men	6180	2.83 (2.5–3.15) P=3e-65	3.13 (1.84–4.42) P=2e-6	0.65
		Women	6462	1.25 (1.16–1.35) P=3e-143	0.257 (-0.408–0.922) P=0.45	0.004
log Very large VLDL [μmol/L]	0.0003	Men	6180	6.42 (5.94–6.9) P=3e-151	7.01 (4.15–9.87) P=2e-6	0.69
		Women	6462	3.00 (2.77–3.23) P=7e-142	0.819 (-0.767–2.4) P=0.31	0.008
log Large VLDL [mmol/L]	0.003	Men	6177	17.5 (16.4–18.7) P=3e-208	17.3 (9.71–24.9) P=8e-6	0.95
		Women	6462	9.19 (9.19–9.19) P=1e-149	3.78 (-0.903–8.45) P=0.11	0.02
VLDL particle size [nm]	0.009	Men	6181	0.113 (0.103–0.122) P=7e-123	0.130 (0.0661–0.194) P=7e-5	0.60
		Women	6462	0.0744 (0.0673–0.0816) P=7e-93	0.0255 (-0.0229–0.0738) P=0.30	0.05
n-6 fatty acids [%]	0.009	Men	6128	-0.321 (-0.361– -0.281); P=3e-55	-0.285 (-0.455– -0.114); P=0.001	0.68
		Women	6400	-0.173 (-0.209– -0.137); P=8e-21	-0.0314 (-0.17–0.107); P=0.66	0.05
Polyunsaturated fatty acids [%]	0.008	Men	6124	-0.316 (-0.367– -0.265); P=4e-34	-0.289 (-0.463– -0.115); P=0.001	0.77
		Women	6395	-0.18 (-0.22– -0.14); P=1.4e-18	-0.0278 (-0.167–0.112); P=0.70	0.04
Saturated fatty acids [mmol/L]	0.002	Men	6390	0.0953 (0.0878–0.103); P=4e-137	0.0736 (0.0216–0.126); P=0.006	0.42
		Women	6122	0.0438 (0.0372–0.0503); P=3e-39	0.0172 (-0.0261–0.0606); P=0.43	0.24
Saturated fatty acids [%]	4e-8	Men	6400	0.126 (0.0626–0.189); P=9e-5	0.222 (-0.0873–0.532); P=0.16	0.55
		Women	6125	0.0104 (-0.0164–0.0373); P=0.45	-0.0961 (-0.253–0.061); P=0.23	0.19
Glutamine [μmol/L]	0.02	Men	6173	-3.99 (-6.1– -1.87); P=0.0002	-3.12 (-8.33–2.09); P=0.24	0.76
		Women	6444	-1.57 (-2.29– -0.848); P=2e-5	2.25 (-1.25–5.74); P=0.21	0.04
Glycine [μmol/L]	0.03	Men	6151	-1.85 (-2.48– -1.22); P=8e-9	-0.956 (-3.42–1.51); P=0.45	0.49
		Women	6427	-0.128 (-0.751–0.496); P=0.69	2.69 (-0.349–5.73); P=0.08	0.08
Homocysteine [μmol/l]	0.0008	Men	1716	0.00713 (-0.0317–0.046); P=0.72	-0.164 (-0.437–0.109); P=0.24	0.22
		Women	2013	0.0345 (0.0068–0.0623);P=0.015	0.166 (-0.191–0.522); P=0.36	0.47
Isoleucine [μmol/L]	0.01	Men	6176	1.51 (1.38–1.63); P=9e-116	1.53 (0.778–2.29); P=7e-5	0.94
		Women	6452	0.883 (0.729–1.04); P=3e-29	0.686 (0.199–1.17); P=0.006	0.45
log Acetoacetate [μmol/L]	0.02	Men	6118	-0.0112 (-0.0151– -0.00719) P=4e-8	-0.0238 (-0.0506–0.00307) P=0.08	0.36
		Women	6407	-0.00483 (-0.00842– -0.00123) P=0.009	0.0122 (-0.0125–0.0368) P=0.33	0.18
log beta-hydroxybutyrate [μmol/L]	4e-5	Men	6123	-0.0168 (-0.0212– -0.0124) P=8e-14	-0.0318 (-0.0588– -0.00485) P=0.02	0.28
		Women	6387	-0.00643 (-0.0102– -0.00265) P=0.0009	0.00983 (-0.0159–0.0355) P=0.45	0.22
Creatinine [μmol/L]	0.03	Men	6161	0.0975 (-0.122–0.317); P=0.38	-0.433 (-1.06–0.193) P=0.18	0.12
		Women	6434	-0.035 (-0.18–0.11) P=0.64	0.0684 (-0.37–0.507) P=0.76	0.66
log Insulin [IU/L]	0.0006	Men	5952	0.0676 (0.0576–0.0775) P=2e-40	0.0548 (0.0315–0.0781) P=4e-6	0.32
		Women	6310	0.054 (0.0498–0.0583) P=1e-136	0.0161 (-0.00369–0.0359) P=0.11	0.0002
Diastolic blood pressure [mmHg]	0.01	Men	6183	0.986 (0.897–1.07) P=1.2e-105	1.36 (0.844–1.87) P=2e-7	0.16
		Women	6444	0.655 (0.533–0.776) P=3e-26	0.865 (0.0861–1.64) P=0.03	0.60

Association magnitudes and causal effect estimates in absolute concentration units.
* Test of difference between cross-sectional association magnitude and causal effect estimate, with P-value calculated using a *Z*-statistic.
