# Supplementary material for: Metabolic Signatures of Adiposity in Young Adults: Mendelian Randomization Analysis and Effects of Weight Change
Source: PLoS Med. 2014 Dec 9;11(12):e1001765. doi: 10.1371/journal.pmed.1001765 (PMC4260795; doi:10.1371/journal.pmed.1001765)
Supplement: Table S5 — Metabolite changes paralleled by weight loss and weight gain during 6-y follow-up in absolute concentration units. (PDF) [file pmed.1001765.s009.pdf]

Table S5: Metabolite changes paralleled by weight loss and weight gain during 6-y follow-up in absolute concentration units.

Metabolite measure [unit]	6–10% weight loss Median [95% CI]	3–6% weight loss Median [95% CI]	3–6% weight gain Median [95% CI]	6–10% weight gain Median [95% CI]
Extremely large VLDL [µmol/L]	-0.00242 [-0.00342- -0.00221]	-0.00221 [-0.00292- -0.00199]	0.00176 [-0.000495-0.00378]	0.00208 [0.000739-0.00557]
Very large VLDL [µmol/L]	-0.00541 [-0.013- -0.00134]	-0.0065 [-0.00964- -0.00134]	0.00315 [-0.00134-0.012]	0.0132 [0.0045-0.0206]
Large VLDL [µmol/L]	-0.03 [-0.0514- -0.0128]	-0.0239 [-0.0369- -0.00931]	0.0326 [0.00279-0.0447]	0.0444 [0.0175-0.0718]
log Medium VLDL [mmol/L]	-0.051 [-0.0806- -0.0216]	-0.0299 [-0.0608- -0.00228]	0.0338 [-0.00276-0.0586]	0.0593 [0.0432-0.0761]
Small VLDL [mmol/L]	-0.0616 [-0.105- -0.0305]	-0.0391 [-0.0639- -0.0154]	0.0449 [0.00976-0.0657]	0.0541 [0.0276-0.106]
Very small VLDL [mmol/L]	-0.044 [-0.0716- -0.0191]	-0.0135 [-0.0318- -0.000406]	0.0143 [-0.00501-0.0331]	0.0358 [0.0183-0.0529]
IDL [mmol/L]	-0.0833 [-0.138- -0.0613]	-0.0116 [-0.0434-0.0294]	0.0316 [0.00281-0.0681]	0.0492 [0.0166-0.0778]
Large LDL [mmol/L]	-0.108 [-0.16- -0.0556]	-0.00359 [-0.0706-0.0311]	0.053 [0.0131-0.0985]	0.0506 [-0.00471-0.0874]
Medium LDL [mmol/L]	-0.071 [-0.0988- -0.0327]	-0.0162 [-0.0456-0.0127]	0.0395 [0.0166-0.0664]	0.03 [-0.0128-0.0616]
Small LDL [mmol/L]	-0.0546 [-0.0665- -0.0306]	-0.0142 [-0.0298-0.00166]	0.0275 [0.0165-0.0483]	0.0194 [0.00146-0.0571]
Very large HDL [mmol/L]	0.0715 [0.0306-0.092]	0.0146 [-0.0162-0.029]	-0.000868 [-0.024-0.0254]	-0.0127 [-0.0393-0.0167]
Large HDL [mmol/L]	0.0463 [2.4e-05-0.0887]	0.0441 [0.00166-0.0843]	-0.0453 [-0.0799- -0.00748]	-0.04 [-0.0639-0.000217]
Medium HDL [mmol/L]	-0.0378 [-0.0763- -0.016]	-0.0242 [-0.0487-0.0121]	-0.0107 [-0.0471-0.0613]	0.0285 [-0.00153-0.0597]
Small HDL [mmol/L]	-0.041 [-0.0697- -0.0152]	-0.000858 [-0.0328-0.0229]	0.00971 [-0.0206-0.0395]	-0.00157 [-0.0272-0.039]
VLDL particle size [nm]	-0.324 [-0.435- -0.146]	-0.247 [-0.459- -0.0312]	0.238 [0.107-0.448]	0.335 [0.0978-0.552]
LDL particle size [nm]	0.0325 [0.00743-0.0529]	0.0229 [-0.00292-0.0371]	-0.0414 [-0.0689- -0.0233]	-0.0129 [-0.0318-0.0175]
HDL particle size [nm]	0.0482 [0.0252-0.067]	0.0168 [-0.00304-0.0439]	-0.0173 [-0.0428- -0.000669]	-0.0343 [-0.0524-0.00938]
Total cholesterol [mmol/L]	-0.212 [-0.393- -0.118]	-0.107 [-0.193-0.0237]	0.132 [0.0262-0.209]	0.0861 [0.00245-0.244]
Non-HDL cholesterol [mmol/L]	-0.219 [-0.322- -0.139]	-0.0904 [-0.199-0.000176]	0.126 [0.0326-0.237]	0.115 [0.0505-0.212]
VLDL cholesterol [mmol/L]	-0.0588 [-0.0999- -0.0156]	-0.0419 [-0.0662- -0.00357]	0.024 [-0.0147-0.0555]	0.0472 [-0.0122-0.0923]
IDL cholesterol [mmol/L]	-0.0463 [-0.0792- -0.0137]	-0.00632 [-0.0203-0.00885]	0.00735 [-0.0119-0.0396]	0.0317 [0.0083-0.0432]
LDL cholesterol [mmol/L]	-0.17 [-0.236- -0.0893]	-0.0534 [-0.109-0.0141]	0.0742 [0.021-0.114]	0.0752 [-0.0376-0.126]
HDL cholesterol [mmol/L]	0.0322 [-0.0184-0.0634]	0.0186 [-0.0189-0.0592]	0.00495 [-0.0565-0.0339]	-0.0145 [-0.103-0.0478]
Cholesterol esterification [%]	-0.000185 [-0.00346-0.00293]	-0.00157 [-0.00282-0.00154]	-0.000916 [-0.00334-0.00284]	0.000919 [-0.00227-0.0042]
Apolipoprotein B [g/l]	-0.07 [-0.0974- -0.0461]	-0.03 [-0.0487- -0.00969]	0.0275 [0.00555-0.0578]	0.0528 [0.0263-0.0727]
Apolipoprotein A1 [g/l]	0.000112 [-0.0558-0.016]	-0.0169 [-0.0403-0.00896]	0.0212 [-0.0239-0.0522]	-0.00383 [-0.0484-0.0368]

Metabolite measure [unit]	6–10% weight loss Median [95% CI]	3–6% weight loss Median [95% CI]	3–6% weight gain Median [95% CI]	6–10% weight gain Median [95% CI]
log Triglycerides [mmol/L]	-0.0638 [-0.0966- -0.0357]	-0.0338 [-0.0626- -0.012]	0.0376 [0.00554-0.0636]	0.0784 [0.0477-0.114]
Phosphoglycerides [mmol/L]	-0.0376 [-0.064-0.00187]	-0.0129 [-0.0342-0.0116]	0.000255 [-0.0207-0.0419]	0.0131 [-0.019-0.0484]
Phosphatidylcholines [mmol/L]	-0.0702 [-0.11- -0.0101]	-0.0204 [-0.0725-0.0394]	0.046 [-0.0432-0.0933]	0.0282 [-0.0399-0.0867]
Sphingomyelin [mmol/L]	-0.0106 [-0.0284-0.0094]	0.00247 [-0.0059-0.0134]	0.00478 [-0.0125-0.0147]	-0.00104 [-0.0152-0.00493]
Total fatty acids [mmol/L]	-0.731 [-1.09- -0.352]	-0.328 [-0.55- -0.0261]	0.402 [-0.0851-0.86]	0.526 [0.241-0.994]
Docosahexaenoic acid [mmol/L]	-0.0108 [-0.0213- -0.00193]	-0.00387 [-0.0145-0.00539]	0.0153 [0.00184-0.0257]	0.00146 [-0.00637-0.0113]
Linoleic acid [mmol/L]	-0.117 [-0.182- -0.0548]	-0.0412 [-0.113-0.0283]	0.0681 [-0.0393-0.173]	0.111 [0.0553-0.203]
n-3 fatty acids [mmol/L]	-0.0198 [-0.0383- -0.00278]	-0.0172 [-0.0357-0.00428]	0.0271 [0.00428-0.0519]	0.0126 [-0.00177-0.0471]
n-3 fatty acids [%]	0.0456 [-0.138-0.19]	-0.0239 [-0.144-0.177]	0.00848 [-0.128-0.156]	-0.0226 [-0.233-0.112]
n-6 fatty acids [mmol/L]	-0.152 [-0.255- -0.0333]	-0.0432 [-0.138-0.0387]	0.0834 [-0.0445-0.197]	0.137 [0.0363-0.21]
n-6 fatty acids [%]	1.04 [0.431-2.01]	0.425 [-0.299-1.02]	-0.466 [-1.32-0.513]	-0.646 [-1.35-0.232]
Polyunsaturated fatty acids [mmol/L]	-0.132 [-0.258- -0.0164]	-0.0745 [-0.171-0.0571]	0.129 [-0.0238-0.257]	0.104 [0.0502-0.248]
Polyunsaturated fatty acids [%]	0.0103 [0.000677-0.017]	0.0065 [0.000202-0.0115]	-0.00268 [-0.00986-0.00482]	-0.00479 [-0.0141-0.00377]
Monounsaturated fatty acids [mmol/L]	-0.314 [-0.428- -0.175]	-0.0985 [-0.258-0.0907]	0.129 [0.00119-0.346]	0.192 [0.0779-0.358]
Monounsaturated fatty acids [%]	-0.00648 [-0.0126- -0.000205]	-0.00623 [-0.00952-0.0014]	0.00342 [-0.00217-0.00943]	0.00387 [-0.00235-0.012]
Saturated fatty acids [mmol/L]	-0.245 [-0.387- -0.0822]	-0.146 [-0.219-0.0114]	0.127 [-0.0282-0.281]	0.228 [0.025-0.387]
Saturated fatty acids [%]	0.00102 [-0.00542-0.00608]	-0.00382 [-0.00876- -0.0011]	-0.00423 [-0.00899-0.00252]	0.000785 [-0.00505-0.00934]
Double bonds/ Fatty acid	0.00307 [-0.00508-0.0147]	0.0122 [-0.000349-0.0247]	0.00392 [-0.0149-0.0171]	-0.0177 [-0.0351-0.000948]
Methylene groups/ Fatty acid	-0.0538 [-0.0801- -0.00885]	-0.00512 [-0.0249-0.0361]	-0.000486 [-0.04-0.0427]	0.00781 [-0.0539-0.0778]
Fatty acid chain length	0.0162 [-0.0274-0.0552]	0.0384 [0.0083-0.0757]	0.0115 [-0.0257-0.0405]	-0.0272 [-0.0758-0.0235]
Glucose [mmol/L]	-0.132 [-0.182- -0.0324]	-0.0824 [-0.142-0.0276]	0.0676 [-0.0924-0.133]	0.0176 [-0.102-0.138]
Lactate [mmol/L]	-0.00141 [-0.111-0.0386]	0.0136 [-0.0314-0.109]	0.00859 [-0.0314-0.109]	0.0286 [-0.0164-0.119]
Pyruvate [µmol/l]	-0.00474 [-0.00934- -0.00104]	0.00126 [-0.00224-0.00421]	-0.00104 [-0.00584-0.00676]	0.00396 [-0.00204-0.00716]
Citrate [µmol/l]	0.00486 [0.000158-0.00876]	-0.00114 [-0.00414-0.00386]	0.00186 [-0.00374-0.00456]	0.00386 [-0.00364-0.00686]
Glycerol [µmol/l]	0.00219 [-0.00776-0.00799]	-0.00171 [-0.00841-0.00609]	0.00499 [-0.000711-0.0109]	0.00479 [-0.00111-0.0114]
Alanine [µmol/l]	-0.015 [-0.026- -0.011]	-0.005 [-0.016-0.008]	0.013 [0.002-0.023]	0.0045 [-0.01-0.0155]
Glutamine [µmol/l]	-0.00666 [-0.0166-0.0113]	0.00734 [-0.00966-0.0213]	0.00234 [-0.0117-0.0123]	-0.0292 [-0.0387- -0.0142]

Metabolite measure [unit]	6–10% weight loss Median [95% CI]	3–6% weight loss Median [95% CI]	3–6% weight gain Median [95% CI]	6–10% weight gain Median [95% CI]
Glycine [µmol/l]	4.66e-05 [-0.00795-0.00805]	0.00205 [-0.011-0.01]	4.66e-05 [-0.00945-0.00755]	-0.00995 [-0.019-0.00205]
Histidine [µmol/l]	-0.00317 [-0.00587- -0.000866]	0.000334 [-0.00167-0.00263]	0.00193 [-0.000566-0.00403]	-0.00162 [-0.00427-0.000834]
Isoleucine [µmol/l]	-0.00476 [-0.00661- -0.00191]	-0.000612 [-0.00331-0.00119]	0.00239 [0.000688-0.00399]	0.00339 [0.000388-0.00629]
Leucine [µmol/l]	-0.00575 [-0.00775- -0.00405]	-0.000247 [-0.00305-0.00255]	0.00315 [0.000253-0.0063]	0.0022 [-0.000247-0.00605]
Valine [µmol/l]	-0.00834 [-0.0143- -0.00234]	0.000659 [-0.00634-0.00566]	0.00966 [0.00366-0.0167]	0.00266 [-0.00284-0.00766]
Phenylalanine [µmol/l]	-0.0045 [-0.0058- -0.0022]	-0.001 [-0.0032-0.000696]	0.0022 [0.000396-0.00545]	0.0026 [0.00105-0.0045]
Tyrosine [µmol/l]	-0.00285 [-0.00535- -0.00105]	-0.00155 [-0.00345-0.000847]	0.00265 [0.000847-0.0043]	0.0025 [0.000297-0.00425]
Acetate [µmol/l]	0.000635 [-0.000665-0.00324]	0.000435 [-0.00166-0.00204]	-0.000565 [-0.00216-0.00229]	0.000985 [-0.00221-0.00324]
log Acetoacetate [µmol/l]	0.00296 [-0.11-0.101]	0.0898 [-0.017-0.222]	-0.0425 [-0.151-0.082]	-0.0771 [-0.152-0.0114]
log beta-hydroxy-butyrate [µmol/l]	-0.0406 [-0.14-0.0761]	0.0361 [-0.0396-0.105]	-0.0697 [-0.201-0.0496]	-0.0632 [-0.131-0.0164]
Creatinine [µmol/l]	-0.000763 [-0.00246-0.00104]	0.00114 [-0.000163-0.00344]	0.000637 [-0.000563-0.00214]	-0.00181 [-0.00336-0.000137]
Urea [µmol/l]	-0.00241 [-0.0109-0.00619]	0.00469 [-0.000406-0.0103]	9.36e-05 [-0.00756-0.00829]	0.00649 [-0.00441-0.0105]
Albumin [cu]	-0.00186 [-0.00288-0.000799]	2.39e-05 [-0.000976-0.00112]	-0.000513 [-0.00264-0.001]	-0.00254 [-0.00403- -0.000651]
log C-reactive protein [mg/L]	-0.266 [-0.426- -0.149]	-0.18 [-0.316- -0.061]	0.281 [0.119-0.4]	0.451 [0.306-0.644]
Glycoprotein acetyls [cu]	-0.0922 [-0.122- -0.0522]	-0.0522 [-0.0722- -0.0222]	0.00776 [-0.0321-0.0478]	0.0478 [-0.00224-0.0678]
log gamma-glutamine amino-transferase [U/L]	-0.111 [-0.195- -0.111]	-0.0748 [-0.111- -0.0158]	0.101 [0.0632-0.129]	0.157 [0.043-0.207]
Adiponectin [ug/mL]	0.441 [0.106-1.03]	-0.0325 [-0.469-0.384]	-0.962 [-1.32- -0.175]	-0.774 [-1.16- -0.413]
Testosterone (Men) [nmol/l]	1.43 [0.527-2.43]	1.03 [-0.273-1.63]	-0.823 [-1.57-0.427]	-1.47 [-2.17- -0.773]
log SHBG (men) [nmol/l]	0.101 [0.0641-0.178]	0.063 [0.0277-0.0907]	-0.105 [-0.15- -0.0621]	-0.116 [-0.168- -0.0858]
log Vitamin D [nmol/l]	0.0628 [0.011-0.11]	0.0192 [-0.037-0.0637]	0.0144 [-0.0278-0.0692]	-0.0481 [-0.126-0.0324]
log Insulin [IU/L]	-0.214 [-0.339- -0.0984]	-0.0913 [-0.228-0.00664]	0.146 [0.059-0.228]	0.216 [0.0796-0.35]
Systolic blood pressure [mmHg]	-1.28 [-3.28-0.716]	-1.28 [-2.62-1.38]	0.716 [-0.951-2.05]	2.72 [-0.617-4.05]
Diastolic blood pressure [mmHg]	-1.28 [-3.61-0.392]	-2.28 [-3.61- -0.942]	1.06 [-0.942-2.39]	2.39 [1.06-5.06]

Metabolite changes paralleled by weight loss and weight gain during 6-y follow-up in the four categories of weight change shown in Figure 7 scaled to absolute concentrations units. The metabolite changes are median [95% confidence intervals]. The foure weight change categories are 6–10% weight loss (n=169; mean (SD) loss 5.5±1.1 kg); 3–6% weight loss (3.2±0.9 kg, n=205); 3–6% weight gain (3.2±0.9 kg; n=168); 6–10% weight gain (5.9±1.7 kg; n=138).
