# Supplementary material for: Metabolic Signatures of Adiposity in Young Adults: Mendelian Randomization Analysis and Effects of Weight Change
Source: PLoS Med. 2014 Dec 9;11(12):e1001765. doi: 10.1371/journal.pmed.1001765 (PMC4260795; doi:10.1371/journal.pmed.1001765)
Supplement: Text S1 — Study populations. (PDF) [file pmed.1001765.s010.pdf]

Study populations

The Northern Finland Birth Cohorts (NFBC) of 1986 and 1966 were initiated to study factors affecting preterm birth and subsequent morbidity in the two northernmost provinces in Finland (www.oulu.fi/NFBC). For NFBC1986, the number of deliveries in the birth cohort was 9,362, which was 99% of all the deliveries taking place in the area during the target period (July 1985-June 1986). Data collection in 2001-2002 included clinical examination and serum sampling at age 15-16 for 6,621 adolescent boys and girls; data from this time point are used for the present study [1,2]. Attendees in the 16-year field study (71%) were representative of the original cohort [2]. In total, 5,602 adolescents had a systemic metabolite profile measured, of which 95% of the serum samples were drawn after overnight fasting. Among these individuals, 3,976 study participants had also information available on the gene score for predisposition to elevated BMI from genotyping using the CardioMetabochip.

The NFBC1966 included 12,058 children born into the cohort, comprising 96% of all births during 1966 in the region [3,4]. Data collection in 1997 included clinical examination and serum sampling at age 31 for 6,007 individuals. Data from this time point are analyzed in the present study. Attendees in the 31-year field study (52%) were representative of the original cohort [3]. In total, 5,709 persons had their metabolite profile measured, of which 96% were based on fasting serum samples [5]. Among these, n=4,671 individuals also had information available on the gene score for elevated BMI based on genome-wide arrays and were included in the present study.

For both NFBC studies, blood pressure was measured using a mercury sphygmomanometer. Physical activity index was calculated as metabolic-equivalent-of-task, based on questionnaire data on frequency, intensity, and duration of physical activity as described previously [1,5]. Current smoking and average alcohol usage were assessed from questionnaires (gram/day; NFBC1966 only). Medication for hypertension, diabetes and hypercholesterolemia was also determined by questionnaires. In addition to NMR-based metabolite quantification, plasma levels of the following protein biomarkers were measured by standard clinical assays and analyzed in the present study: C-reactive protein, alanine aminotransferase, gamma-glutamyl aminotransferase, direct bilirubin, and plasma insulin [5,6]. Testosterone and sex-hormone binding globulin were measured by mass spectrometry. Vitamin-D levels were measured for NFBC1966. Informed written consent was obtained from all participants, and the research protocols were approved by the Ethics Committee of Northern Ostrobotnia Hospital District, Finland.

The Cardiovascular Risk in Young Finns Study (YFS) was designed to study associations of childhood risk factors to cardiovascular disease in adulthood (youngfinnsstudy.utu.fi) [7]. The baseline study conducted in 1980 included 3,596 children and adolescents aged 3–18. Data in the cross-sectional analyses in the present study are predominantly from the 2001 survey, which included 2,247 individuals with an overnight fasting metabolite profile (response rate 63%). These individuals were

representative of the baseline cohort [7]. In addition, 288 individuals who only attended the 2007-survey were included in analyses. Genotype information on the gene score for elevated BMI was available for n=2,171 participants based on genome-wide arrays. Longitudinal data on 6-year change in BMI and change in metabolite levels were collected for 1,488 individuals, who participated in both the 2001 and 2007 field studies and were free of medication for metabolic diseases at both timepoints. The longitudinal associations were further assessed for consistency at 10-year follow-up (2001→2011; n=1,372).

Blood pressure was measured using a random-zero sphygmomanometer. Metabolic-equivalent-of-task was used as physical activity index [5]. Current smoking, average alcohol usage (gram/day) and medication for hypertension, diabetes and hypercholesterolemia were assessed by questionnaires. In addition to NMR metabolite profiling, the following plasma biomarkers were measured by standard clinical assays and analyzed in the present study: lipoprotein(a), homocysteine, C-reactive protein, phospholipase A2 activity, alanine aminotransferase, gamma-glutamyl aminotransferase, leptin, adiponectin, vitamin D, and insulin [5,7] Testosterone and sex-hormone binding globulin were measured by mass spectrometry for both women and men in 2001, but only for men in 2007. All participants gave written informed consent, and the study was approved by the ethics committees of each of the five participating medical university study sites in Finland.

The FINRISK 1997 study was conducted to monitor the health of the Finnish population among persons aged 24–74 at recruitment (thl.fi/finriski) [8,9]. In total, 8,444 individuals were recruited to represent the general population of the study areas. Blood pressure was measured and participants filled in questionnaires of smoking status, alcohol usage, physical activity, and medication.[9] In the absence of sufficient information to derive metabolic-equivalent-of-task, a binary physical activity index was defined based on the self-reported level of leisure time physical activity. Metabolite profiling from serum samples were measured for 7,610 individuals, of which 5,804 were free of prevalent cardiometabolic disease or treatment of lipid levels or hypertension. The median fasting time was 5h (interquartile range 4–6h). Only individuals under the age of 40 were included the primary analyses of the present study to minimize the influence of aging and reverse causality from the comorbidities of obesity. Out of 2,128 participants in this age group, genotype information on the gene score was available for n=1,846. The generalizability of the cross-sectional associations to older age groups was examined separately for 3,676 individuals aged 40–74 (Figure 3). In addition to NMR-based metabolite profiling, the following circulating biomarkers were assayed by standard clinical assays and analyzed in the present study: lipoprotein(a), homocysteine, C-reactive protein, phospholipase A2 activity, gamma-glutamyl aminotransferase, leptin, adiponectin, testosterone, vitamin D, and insulin [9]. Participants gave written informed consent and the FINRISK study was approved by the ethical committee of the National Public Health Institute, Helsinki, Finland.

The Pieksämäki study

The generalizability of the cross-sectional and longitudinal results to older age groups was examined in the Pieksämäki population cohort [10,11]. The Pieksämäki cohort consisted of individuals from the town of Pieksämäki, Eastern Finland, born in 1942, 1947, 1952, 1957 and 1962. There were 923 participants in the initial examination in 1997. In the present study, 628 individuals aged 40–57 with metabolite profile quantified and free of baseline medication were analyzed for the generalization of the cross-sectional results to older age groups (Figure 3). Of these, 456 persons had their metabolite profile measured again at a follow-up survey in 2003, which enabled generalization of the longitudinal results to older age (Figure S4). No genotype information was available. In addition to NMR-based metabolite profiling, the following metabolic measures were assayed in the Pieksämäki study: C-reactive protein, adiponectin, insulin, and blood pressure. Participants gave written informed consent and the study protocol was approved by the Ethics Committee of Kuopio University Hospital.

Clinical definitions

Overweight was defined as BMI of 25-29.9 kg/m^2 and obesity as BMI≥30 kg/m^2.

References:

1. Kantomaa MT, Stamatakis E, Kankaanpää A, et al. (2013) Physical activity and obesity mediate the association between childhood motor function and adolescents' academic achievement. Proc Natl Acad Sci U S A 110: 1917–1922.
2. Veltsista A, Laitinen J, Sovio U, Roma E, Järvelin MR, Bakoula C (2010) Relationship between eating behavior, breakfast consumption, and obesity among Finnish and Greek adolescents. J Nutr Educ Behav 42: 417–421.
3. Sabatti C, Service SK, Hartikainen AL, et al. (2009) Genome-wide association analysis of metabolic traits in a birth cohort from a founder population. Nat Genet 41: 35–46.
4. Järvelin MR, Sovio U, King V, et al. (2004) Early life factors and blood pressure at age 31 years in the 1966 northern Finland birth cohort. Hypertension 44: 838–46.
5. Würtz P, Mäkinen VP, Soininen P, et al. (2012) Metabolic signatures of insulin resistance in 7,098 young adults. Diabetes 61: 1372–1380.
6. Chambers JC, Zhang W, Sehmi J, et al. (2011) Genome-wide association study identifies loci influencing concentrations of liver enzymes in plasma. Nat Genet 43:1131–1138.
7. Raitakari OT, Juonala M, Rönnemaa T, et al. (2008) Cohort profile: The cardiovascular risk in young Finns study. Int J Epidemiol 37:1220–1226.
8. Vartiainen E, Laatikainen T, Peltonen M, et al. (2010) Thirty-five-year trends in cardiovascular risk factors in Finland. Int J Epidemiol 39:504–518.
9. Blankenberg S, Zeller T, Saarela O, et al. (2010) Contribution of 30 biomarkers to 10-year cardiovascular risk estimation in 2 population cohorts: the MONICA, risk, genetics, archiving, and monograph (MORGAM) biomarker project. Circulation 121:2388–2397.
10. Würtz P, Tiainen M, Mäkinen VP, et al. (2012) Circulating metabolite predictors of glycemia in middle-aged men and women. Diabetes Care 35:1749–1756.
11. Mäntyselkä P, Kautiainen H, Saltevo J, et al. (2012) Weight change and lipoprotein particle concentration and particle size: a cohort study with 6.5-year follow-up. Atherosclerosis 223:239–43.
